# Supplementary material for: Nonlinear plasmon-exciton coupling enhances sum-frequency generation from a hybrid metal/semiconductor nanostructure
Source: Nat Commun. 2020 Mar 19;11:1464. doi: 10.1038/s41467-020-15232-w (PMC7081225; doi:10.1038/s41467-020-15232-w)
Supplement: Supplementary file 1 — Supplementary Information [file 41467_2020_15232_MOESM1_ESM.pdf]

Supplementary information for

‘Nonlinear plasmon-exciton coupling enhances sum-frequency generation from a hybrid metal/semiconductor nanostructure’

Zhong et al.

Supplementary Note 1: Characterization of nanosponges

This section presents additional SEM and TEM images, and scattering spectra that characterize bare gold and hybrid Au/ZnO nanosponges.

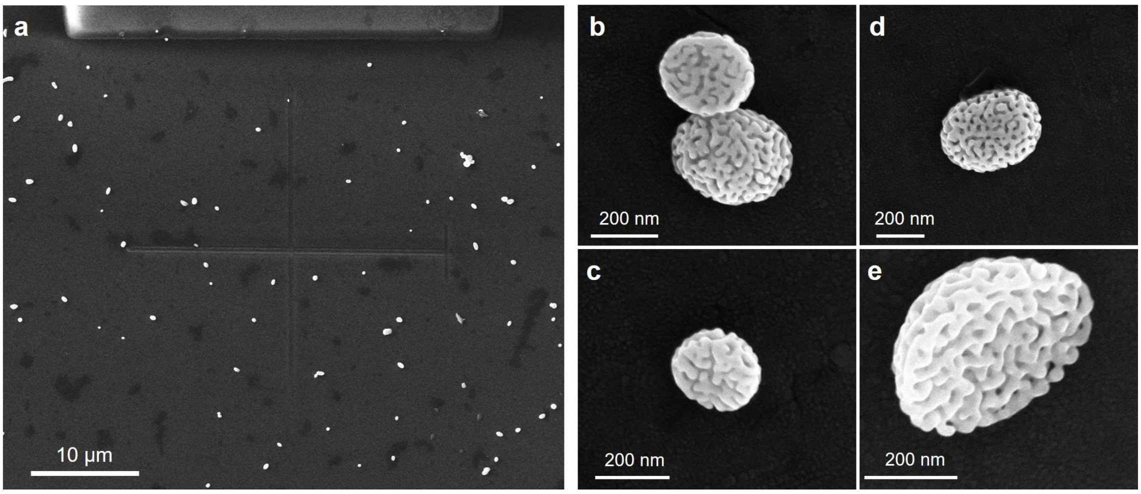

Supplementary Fig. 1 | SEM images of bare Au nanosponges. (a) An overview of the sample. (b-e) Zoom-in views of single nanosponges, which are half-spheres of round or elliptical shapes with diameters of about 300 nm. The nanosponges were transferred from SiO_2/Si substrate to ITO surface, resulting in different orientations (flat lying or flipped) of particles on the surface. The images clearly illustrate that nanosponges are perforated with ~10-20 nm sized nanopores throughout the entire particle.[1-3]

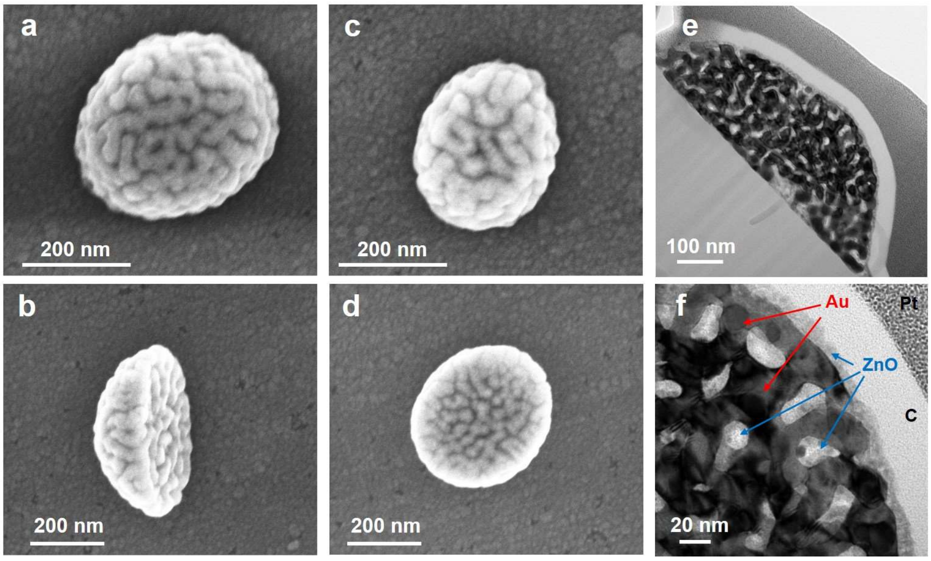

Supplementary Fig. 2 | SEM and TEM images of hybrid Au/ZnO nanosponges. (a-d) SEM images of several hybrid nanosponges. The nanopores have been infiltrated with ZnO, by comparing to the images of bare Au nanosponges (Supplementary Fig. 1). (e-f) Cross-sectional TEM images of a hybrid nanosponge prepared by first depositing protecting carbon (C) and platinum (Pt) layers and then cut by a focused ion beam. A 10-nm think ZnO layer has been deposited on the surface and in the interior of the nanosponges.

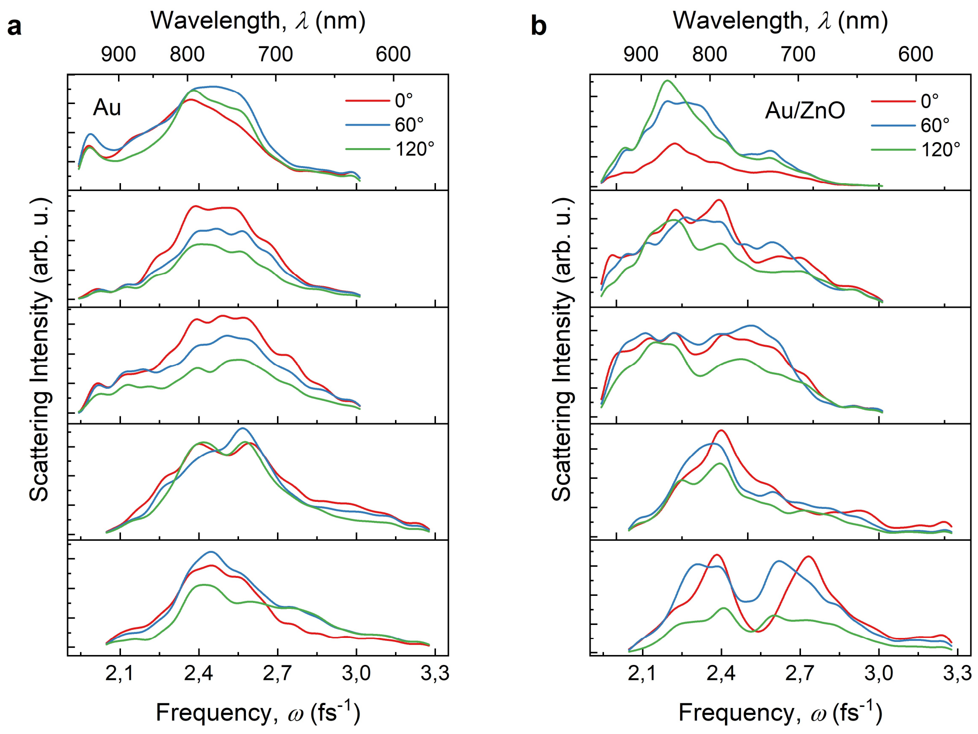

Supplementary Fig. 3 | Single particle dark-field white-light scattering spectra. (a) Excitation-polarization resolved single particle scattering spectra of five bare Au nanosponges. (b) Similar spectra for five Au/ZnO hybrid nanosponges. For each particle, scattering spectra at three different excitation-polarization angles are compared. The spectra show broad resonances ranging from 600 nm to 950 nm superimposed with some random spectral modulations (multiple peak maximums), depending on the particle size and shape. The results are consistent with previous reports.[4-6] The spectra reflect the superposition of the broadband plasmonic dipole mode of the particle and the localized plasmonic modes of multiple hot spots with different resonance frequencies.

Supplementary Note 2: Pulse characterization

To characterize the electric field of the laser pulse, a 15-µm thick beta barium borate (BBO) crystal is used as the sample. Supplementary Fig. 4a shows the measured IFRAC trace from the BBO, and the Fourier-transformed DC and FM bands are shown in Supplementary Fig. 4b and 3c, respectively. To compensate the limited bandwidth of the BBO with finite thickness, we apply a frequency marginal correction to the measured IFRAC trace using a typical sinc^2-shaped phase-matching function.[7-9] A pulse retrieval algorithm[7, 10] is used to retrieval the electric field of the pulse, using the DC components of the IFRAC trace. The retrieved electric field $E_{\mathrm{L}}(t)$ of the few-cycle pulse is depicted in Supplementary Fig. 4e, with a pulse duration (full width at half maximum of the intensity profile, $I(t) = |E_{\mathrm{L}}(t)\,|^2$ of ~8 fs. The retrieved spectrum matches well to the independently measured spectrum in a spectrometer (Supplementary Fig. 4f), reproducing well the peak structures of the laser profile. The slight reduction of the intensity at short and long wavelength range may result from the limited bandwidth of the BBO. We note that the modulated structure of laser is critical for interpreting and simulating the results. The spectral phase is generally flat over the central wavelength range. The simulated IAC trace matches well to the measured one (Supplementary Fig. 4d), indicating the pulse has been well-retrieved. The retrieved laser field $E_{\mathrm{L}}(t)$ is used as the far-field excitation source in simulations to model the nonlinear emission from nanosponges.

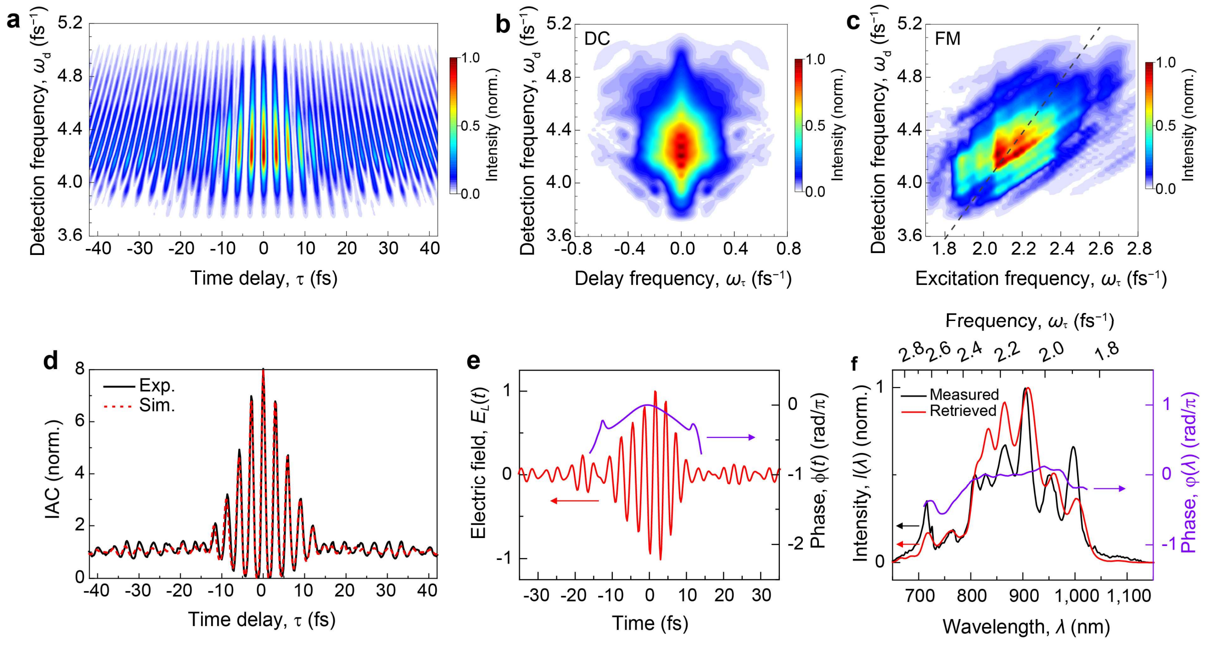

Supplementary Fig. 4 | Pulse characterization. (a) IFRAC trace of BBO. (b) Fourier-transformed DC band of the IFRAC. (c) Fourier-transformed FM band of the IFRAC. (d) Spectrally integrated interferometric autocorrelation (IAC) of the IFRAC (black), matching well to the simulated IAC using the retrieved laser field (dashed red). (e) Retrieved electric field and temporal phase of the pulse. (f) Retrieved spectrum and spectral phase of the pulse. An independently measured laser spectrum (black) is shown for comparison, indicating that the pulse profile has been well-retrieved.

Supplementary Note 3: Excitation-polarization-resolved optical nonlinear emission

This section gives more detailed information and data on the polarization-dependent measurements of bare Au and hybrid Au/ZnO nanosponges.

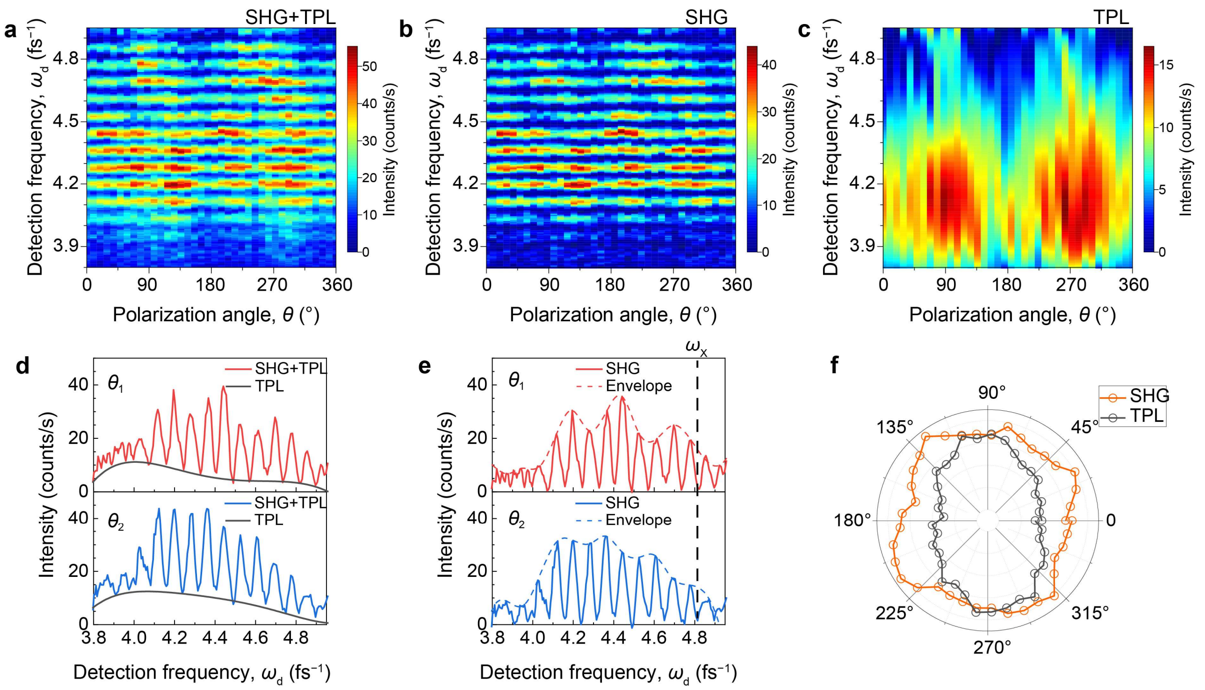

Supplementary Fig. 5 | Excitation-polarization dependent nonlinear emission from the bare gold nanosponge. (a) Colour-code image of the nonlinear emission spectra as a function of the polarization angle (θ) of the linearly polarized incident pulse, at a fixed inter-pulse delay $\tau \sim 75$ fs. The coherent second harmonic (SH) emission (panel b) and incoherent two-photon photoluminescence (TPL) emission (panel c) are separated from the raw data in (a). (d) Nonlinear emission spectrum at $\theta_1 = 175°$ and $\theta_2 = 320°$, which contains both incoherent TPL background (gray curves) and coherent SH characterized by spectral fringes resulting from interference of the nonlinear emission excited by phase-locked pulse pair.[11, 12] For every spectrum, the TPL and SH can be separated by Fourier filtering, giving the results shown in (b) and (c). (e) Coherent SH after filtering TPL from the raw spectrum for the spectra shown in (d). The dashed lines are the spectral envelope. The vertical dashed line marks the exciton frequency at $\omega_X = 4.8$ fs^{-1}. (f) Polar plot of the spectrally integrated intensities of SH and TPL from the data in (b) and (c), indicating TPL emission is dominated by the dipolar mode of the whole particle whereas SH emission comes from multiple hot spot modes with random orientations of polarization.

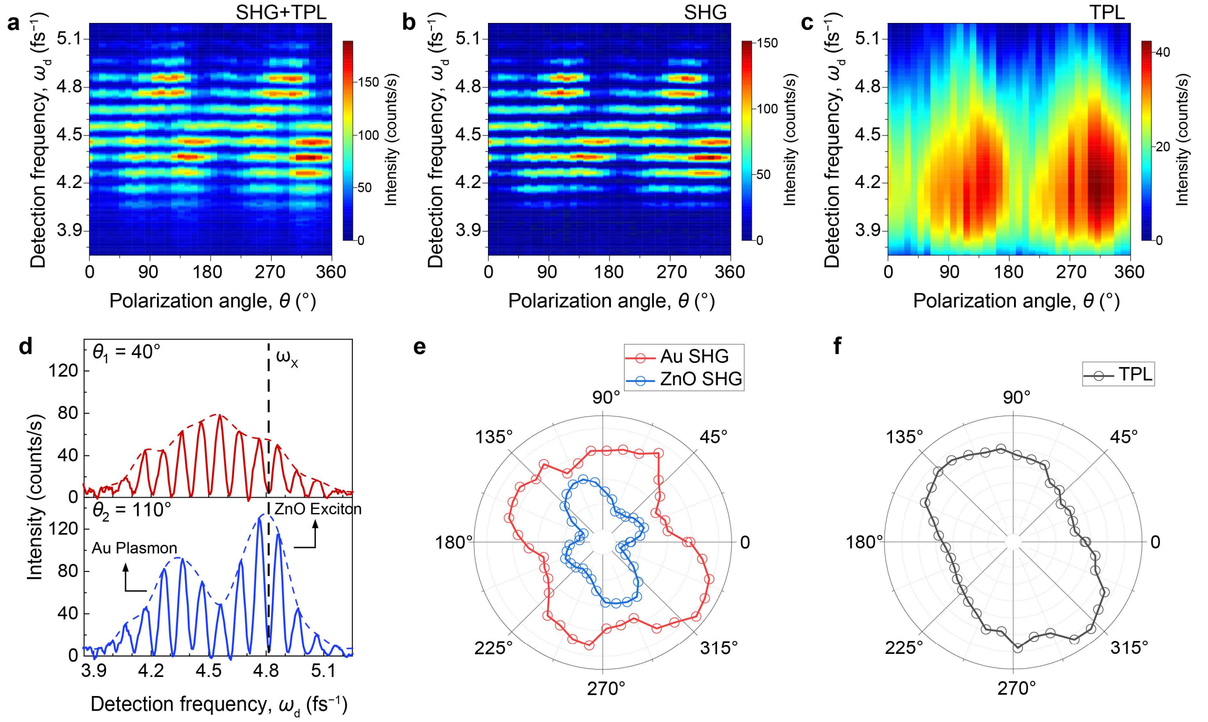

Supplementary Fig. 6 | Excitation-polarization dependent nonlinear emission of the hybrid Au/ZnO nanosponge. (a) Colour-code image of the nonlinear emission spectra as a function of polarization angle (θ) of the incident pulse, recorded at a fixed inter-pulse delay $\tau \sim 63$ fs. The coherent SH emission (b) and incoherent TPL emission (c) are separated from the raw data in (a). (d) SH spectra at two different polarization angles (after subtraction of TPL from the raw spectra as discussed in Supplementary Fig. 5). A distinctly enhanced emission near the ZnO exciton frequency $\omega_X = 4.8$ fs^{-1} is observed, which is about eight times stronger than that of bare gold nanosponges at the same frequency shown in Supplementary Fig. 5e. (e) Polar plot of the spectrally integrated intensities of SH recorded in two different spectral regions: Au SH: 3.8–4.6 fs^{-1} (red circles); ZnO SH: 4.6–5.1 fs^{-1} (blue circles). Multipolar radiation patterns are also observed. (f) Polar plot of the spectrally integrated TPL intensities (3.8–5.1 fs^{-1}) from the data in (c). The emission is mainly dominated by the dipolar mode of the particle.

Supplementary Note 4: Incoherent TPL signal in IFRAC

We have shown in Supplementary Fig. 5 that incoherent TPL and coherent SH can be separated because the latter is characterized by spectral interference fringes due to its coherent nature.[11, 12] For the data from the bare gold nanosponge in Fig. 1b of the main text, the separation of TPL and SH is shown Supplementary Fig. 7 (a-c). We perform this separation only for large inter-pulse time delays to have a sufficient number of fringes with good contrast. The SH signal (Supplementary Fig. 7b) shows detection-frequency dependent fringes while the TPL signal appears as a detection-frequency independent modulation of the IFRAC signal along τ (Supplementary Fig. 7c). In the Fourier-transformed FM band, the SH (or SF) signal distributes along the diagonal line with a slope of 2, showing excitation-emission frequency correlations. In contrast, the TPL signal appears in the form of vertical stripes in the FM band signal without excitation-emission frequency correlations.[11, 12] (Supplementary Fig. 7d). This can most clearly be seen for low ω_d in the data of bare gold nanosponges, as marked by red dashed lines in Supplementary Fig. 7d. In the hybrid nanosponge, the relative contribution of the TPL signal to the FM-band is reduced, which might be explained by the enhanced SH in the hybrid nanosponge. Even though TPL is incoherent and does not show interference fringes in spectral

domain, it is modulated as a function of τ because of the periodic modulation of excited carrier population by tuning τ.[12]

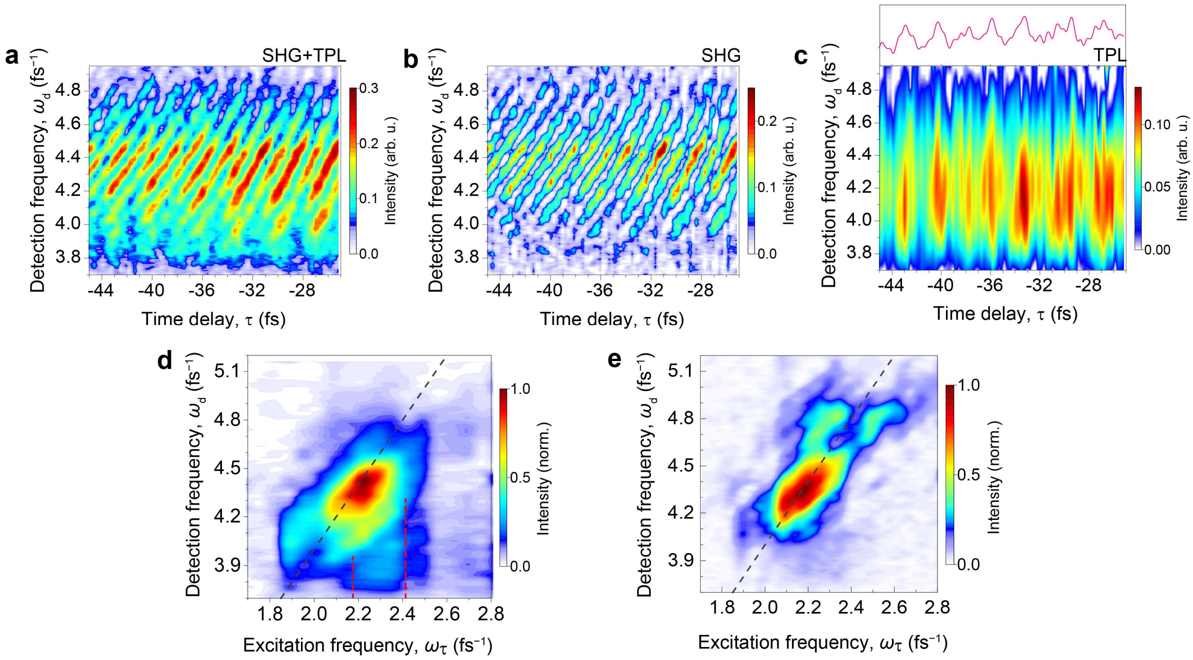

Supplementary Fig. 7 | Separating TPL signal in IFRAC. (a) IFRAC trace of the bare gold nanosponge shown in Fig. 1b of the main text for τ between –45 and –25 fs. The coherent SH signal is superimposed on an incoherent TPL background signal. (b) IFRAC of the pure SH signal. (c) IFRAC of the TPL signal. SH and TPL signals are separated by Fourier filtering as described in Supplementary Fig. 5. The top inset in (c) is the spectrally integrated intensity showing modulations as a function of τ with a constant period. (d) FM band of the IFRAC trace of the bare gold nanosponge shown in Fig. 1b in the main text. The coherent SH signal distributes along the diagonal line with a slope of 2, showing the correlation between emission and excitation frequencies. The TPL signal shows up as an excitation-frequency independent signal, as seen for low detection frequencies in the region marked by the red dashed lines. (e) FM band of the hybrid nanosponge shown in Fig. 4a of the main text. In comparison to the bare Au sponge, the TPL contribution is much reduced.

Supplementary Note 5: Analytical model of IFRAC for a single Lorentzian oscillator

Time-domain IFRAC trace

Here, we discuss the IFRAC signals that result from a single Lorentzian oscillator with resonant angular frequency ω_0 and dephasing rate γ_0, that is impulsively excited by an infinitely short laser pulse $E_{\mathrm{L}}(t)=\delta(t)$. The response of the Lorentzian oscillator is taken as $r(t)=\Theta(t)\sin(\omega_0 t)\mathrm{e}^{-\gamma_0 t}$ with $\Theta(t)$ being the Heaviside function. This gives the frequency domain response function

$$\tilde{r}(\omega)=\int_0^\infty r(t)\mathrm{e}^{\mathrm{i}\omega t}dt=\frac{-1}{2}\left(\frac{1}{\omega-\omega_0+\mathrm{i}\gamma_0}-\frac{1}{\omega+\omega_0+\mathrm{i}\gamma_0}\right) \tag{1}$$

Subject to the excitation of laser pulse $E_L(t)$, the local surface plasmonic field is given as the linear convolution, $E_P(t)=\int_{-\infty}^{t} r(t-t')E_L(t')dt'$, which generates 2nd-order nonlinear field $E_{NL}(t)=\left[E_P(t)\right]^2$. To allow for an analytical expression, the excitation is first taken as a δ-pulse, thus $E_P(t) = r(t)$. Assuming a frequency-independent 2nd-order susceptibility $\chi^{(2)}$, the IFRAC signal at detection frequency ω_d is resolved in a spectrometer and recorded as a function of inter-pulse delay τ, $I_{IF}(\omega_d,\tau)=\left|\int_0^{\infty} E_{NL}(t,\tau)e^{i\omega_d t}dt\right|^2$. Throughout the derivation, we assume $\tau>0$ and the two pulses have the same amplitude. The 2nd-order nonlinear field

$$
\begin{aligned}
E_{NL}(t,\tau)&=(E_P(t)+E_P(t-\tau))^2\\
&=\left(\frac{\Theta(t)}{2i}\left(e^{i\omega_0 t}e^{-\gamma_0 t}-e^{-i\omega_0 t}e^{-\gamma_0 t}\right)+\frac{\Theta(t-\tau)}{2i}\left(e^{i\omega_0(t-\tau)}e^{-\gamma_0(t-\tau)}-e^{-i\omega_0(t-\tau)}e^{-\gamma_0(t-\tau)}\right)\right)^2
\end{aligned}
\tag{2}
$$

gives the Fourier transform

$$
\begin{aligned}
\tilde{E}_{NL}(\omega_d,\tau)&=\int_{-\infty}^{\infty}E_{NL}(t,\tau)e^{i\omega_d t}dt\\
&=-\frac{i}{4}\left(\frac{1+e^{i\omega_d\tau}+2e^{i(\omega_d+\omega_0)\tau}e^{-\gamma_0\tau}}{\omega_d+2\omega_0+i2\gamma_0}-\frac{2+2e^{i\omega_d\tau}+2e^{i(\omega_d+\omega_0)\tau}e^{-\gamma_0\tau}+2e^{i(\omega_d-\omega_0)\tau}e^{-\gamma_0\tau}}{\omega_d+i2\gamma_0}+\frac{1+e^{i\omega_d\tau}+2e^{i(\omega_d-\omega_0)\tau}e^{-\gamma_0\tau}}{\omega_d-2\omega_0+i2\gamma_0}\right)
\end{aligned}
\tag{3}
$$

which contains the second harmonic (SH, term 1 and 3) and optical rectification (OR, term 2) signals. We keep only the SH term at positive frequency that is detected in a spectrometer,

$$\tilde{E}_{NL}(\omega_d,\tau)=-\frac{i}{4}\left(\frac{1+e^{i\omega_d\tau}+2e^{i(\omega_d-\omega_0)\tau}e^{-\gamma_0\tau}}{\omega_d-2\omega_0+i2\gamma_0}\right). \tag{4}$$

The IFRAC intensity follows as

$$I_{IF}(\omega_d,\tau)=\left|\tilde{E}_{NL}(\omega_d,\tau)\right|^2=\frac{1}{16}\frac{\left(2+4e^{-2\gamma_0\tau}+2\cos(\omega_d\tau)+4\cos(\omega_0\tau)e^{-\gamma_0\tau}+4\cos((\omega_d-\omega_0)\tau)e^{-\gamma_0\tau}\right)}{(\omega_d-2\omega_0)^2+4\gamma_0^2}. \tag{5}$$

This shows an IFRAC trace with the spectral envelope in ω_d axis given by a Lorentzian line shape function $I_{SH}(\omega_d,2\omega_0,2\gamma_0)=\frac{1}{(\omega_d-2\omega_0)^2+4\gamma_0^2}$ that is independent on the time delay τ. Their intensity, however, is periodically modulated as a function of τ, as seen from the numerator of Eq. (5). The IFRAC trace $I_{IF}(\omega_d,\tau)$ is symmetric along the delay axis τ. A numerical simulation of IFRAC trace is shown in Supplementary Fig. 8a.

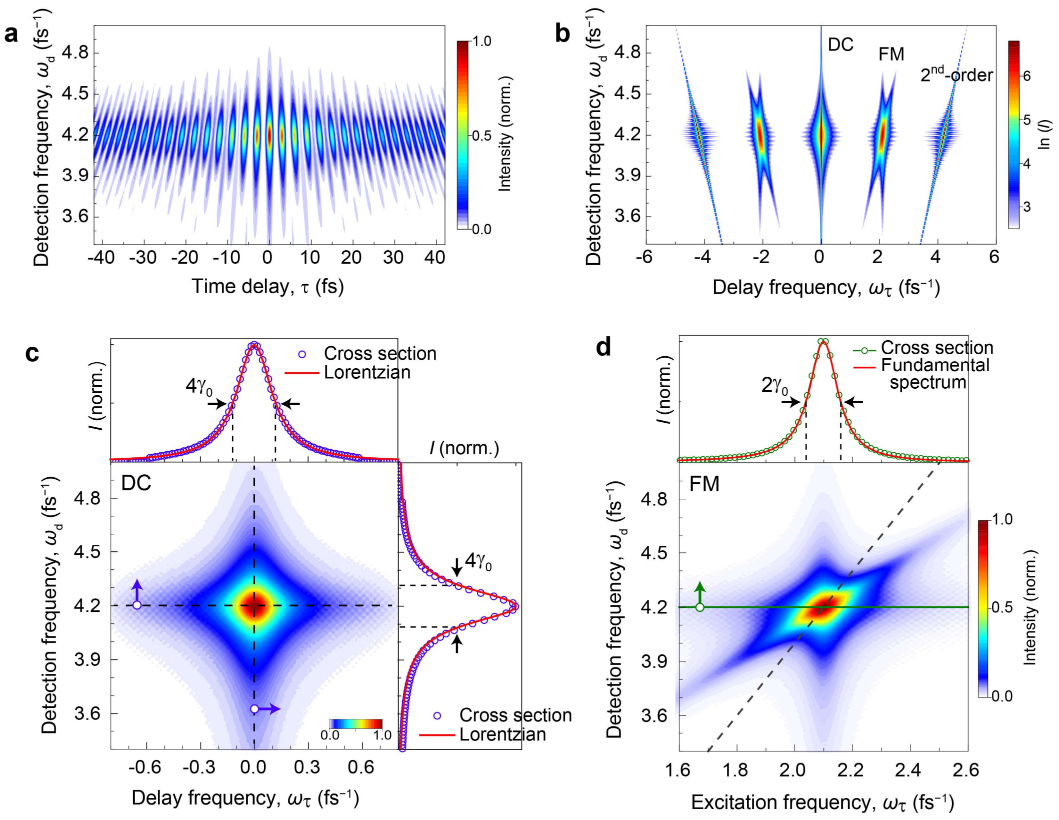

Supplementary Fig. 8 | Numerical simulation of the IFRAC trace of a single Lorentzian oscillator. (a) Simulated IFRAC trace of a Lorentzian oscillator with ω_0 = 2.1 fs^{-1} and γ_0 = 0.06 fs^{-1} excited by δ-function optical pulses. (b) Fourier transform of the IFRAC trace along τ, showing the DC, FM, and 2nd-order bands. (c) The DC band shows a peak at $\omega_d = 2\omega_0$ with symmetric line shapes along both ω_τ and ω_d. Horizonal and vertical cross sections along the dashed lines are given in the insets (blue circles) and fitted to Lorentzian line shapes (red lines, Eq. (8)) with a width of $4\gamma_0$. (d) FM band of the IFRAC trace. The signal is symmetric with respect to the diagonal dashed line with a slope of 2, in the sense that $I_{IF}(\omega_d,\omega_\tau=\omega_d/2-\Delta\omega)=I_{IF}(\omega_d,\omega_\tau=\omega_d/2+\Delta\omega)$ from Eq. (12). Top inset: the cross-sectional spectrum (green circles) taken $\omega_d = 2\omega_0$ (green line in the image), matches to the linear spectrum of the Lorentzian oscillator (red line) according to Eq. (13).

Fourier transform of IFRAC trace along the time delay axis

The Fourier transform of IFRAC intensity signal in Eq. (5) along τ axis gives rise to a new delay frequency ω_τ, for which we obtain, by noting that $I_{IF}(\omega_d,\tau)=I_{IF}(\omega_d,-\tau)$,

$$\begin{aligned}
&\tilde{I}_{IF}(\omega_d,\omega_\tau)=\int_{-\infty}^{\infty} I_{IF}(\omega_d,\tau)e^{i\omega_\tau\tau}d\tau \\
&=\frac{1}{16}\frac{1}{(\omega_d-2\omega_0)^2+4\gamma_0^2}\int_{-\infty}^{\infty}\left(2+4e^{-2\gamma_0|\tau|}+2\cos(\omega_d\tau)+4\cos(\omega_0\tau)e^{-\gamma_0|\tau|}+4\cos\left((\omega_d-\omega_0)\tau\right)e^{-\gamma_0|\tau|}\right)e^{i\omega_\tau\tau}d\tau \qquad (6)\\
&=\frac{1}{16}\frac{1}{(\omega_d-2\omega_0)^2+4\gamma_0^2}\hat{I}_{IF}(\omega_d,\omega_\tau)
\end{aligned}$$

with

$$\hat{I}_{\rm IF}(\omega_{\rm d},\omega_\tau)=\begin{Bmatrix} 4\pi\delta(\omega_\tau)+\dfrac{16\gamma_0}{\omega_\tau^2+4\gamma_0^2}+2\pi\delta(\omega_\tau+\omega_{\rm d})+2\pi\delta(\omega_\tau-\omega_{\rm d})+ \\ \dfrac{4\gamma_0}{(\omega_\tau+\omega_0)^2+\gamma_0^2}+\dfrac{4\gamma_0}{(\omega_\tau-\omega_0)^2+\gamma_0^2}+\dfrac{4\gamma_0}{(\omega_\tau+(\omega_{\rm d}-\omega_0))^2+\gamma_0^2}+\dfrac{4\gamma_0}{(\omega_\tau-(\omega_{\rm d}-\omega_0))^2+\gamma_0^2} \end{Bmatrix}. \quad (7)$$

A representation of the IFRAC signal $\tilde{I}_{\rm IF}(\omega_{\rm d},\omega_\tau)$ along the detection and delay frequency axis, represents a two-dimensional (2D) optical spectrum as shown in Supplementary Fig. 8b. For a purely 2$^{\rm nd}$-order nonlinear process, this 2D spectrum contains five distinct bands at $\omega_\tau = \pm n(\omega_{\rm d}/2)$ with n =0,1,2 corresponding to zeroth-order (DC), fundamental (FM), and 2$^{\rm nd}$-order bands, respectively. Note that in all figures, we shall refer to ω_τ as delay frequency in DC band and excitation frequency in FM band in accordance with general usage.

We now examine each of the different terms of the IFRAC trace in Eqs. (5) and (7) in the time (τ) and frequency (ω_τ) domains.

(1) The first term in Eq. (5) is a constant signal in time domain. In the frequency domain, it becomes a δ−function $\hat{I}_{\rm IF,1}(\omega_\tau)=\int_{-\infty}^{\infty}2e^{i\omega_\tau\tau}d\tau=4\pi\delta(\omega_\tau)$. It contributes to the DC band but carries no information about the studied system. This term is removed in data analysis.

(2) The exponentially decaying second term, $4\,e^{-2\gamma_0\tau}$, yields in the frequency domain,

$$\tilde{I}_{\rm IF}^{\rm DC}(\omega_{\rm d},\omega_\tau)=\frac{1}{(\omega_{\rm d}-2\omega_0)^2+4\gamma_0{}^2}\frac{\gamma_0}{\omega_\tau^2+4\gamma_0^2}. \quad (8)$$

This suggests that the spectra along the $\omega_{\rm d}$ axis have a line shape that is resonant at the SH of the Lorentz oscillator, $\omega_{\rm d}=2\omega_0$, and has a width of $4\gamma_0$ (full width at half maximum, FWHM). Along ω_τ axis, the peak is resonant at $\omega_\tau = 0$ and has the same width of $4\gamma_0$. Therefore, for SH generation from a single, homogeneously broadened Lorentzian oscillator, the line shape of the DC band is symmetric along $\omega_{\rm d}$ and ω_τ. This can be seen in the simulation result shown in Supplementary Fig. 8c. Both of the above two terms appear in DC band, which can be first separated from the whole Fourier-transformed IFRAC bands by applying a super-Gaussian filter.[7] To isolate the 2$^{\rm nd}$ DC term, the DC band is transformed back to time domain and a constant term is subtracted. The resulting signal is then back transformed to the frequency domain and the pure DC band is obtained, as shown in Supplementary Fig. 8c.

(3) For the third term, $2\cos(\omega_{\rm d}\tau)$, in Eq. (5) we obtain

$$\hat{I}_{\rm IF,3}(\omega_\tau)=\int_{-\infty}^{\infty}(e^{-i\omega_{\rm d}\tau}+e^{i\omega_{\rm d}\tau})e^{i\omega_\tau\tau}d\tau=2\pi\delta\left((\omega_\tau+\omega_{\rm d})+(\omega_\tau-\omega_{\rm d})\right). \quad (9)$$

In the time domain, this signal is modulated with a frequency of $\omega_{\rm d}$, that is, the SH frequency and shows no damping. In the frequency domain, it appears as a delta function at $\omega_\tau = \pm\,\omega_{\rm d}$. It constitutes the 2$^{\rm nd}$-order band and carriers no information about optical response of the system since it only reflects the interference of a specific frequency component of the two time-delayed pulses.

(4) The fourth term, $4\cos(\omega_0\tau)e^{-\gamma_0\tau}$, leads to

$$\hat{I}_{\mathrm{IF},4}(\omega_\tau)=4\int_{-\infty}^{\infty}\mathrm{e}^{-\gamma_0|\tau|}\cos(\omega_0\tau)\mathrm{e}^{\mathrm{i}\omega_\tau\tau}d\tau=\frac{4\gamma_0}{(\omega_\tau+\omega_0)^2+\gamma_0^2}+\frac{4\gamma_0}{(\omega_\tau-\omega_0)^2+\gamma_0^2}. \tag{10}$$

(5) The fifth term in Eq. (5), $4\cos((\omega_{\mathrm{d}}-\omega_0)\tau)\mathrm{e}^{-\gamma_0\tau}$, gives

$$\hat{I}_{\mathrm{IF},5}(\omega_\tau)=4\int_{-\infty}^{\infty}\mathrm{e}^{-\gamma_0|\tau|}\cos((\omega_{\mathrm{d}}-\omega_0)\tau)\mathrm{e}^{\mathrm{i}\omega_\tau\tau}d\tau=\frac{4\gamma_0}{(\omega_\tau+(\omega_{\mathrm{d}}-\omega_0))^2+\gamma_0^2}+\frac{4\gamma_0}{(\omega_\tau-(\omega_{\mathrm{d}}-\omega_0))^2+\gamma_0^2}. \tag{11}$$

The 4th and 5th terms are similar, and both appear in the FM band. Their difference lies in the different resonance positions along ω_τ. While the 4th term peaks at ω_0 for all detection frequency, the resonance of the 5th term lies at $\omega_{\mathrm{d}}-\omega_0$. Supplementary Fig. 8d shows the FM band of the simulated IFRAC trace.

In total, keeping only terms at positive ω_τ, the full expression for the FM band is

$$\tilde{I}_{\mathrm{IF}}^{\mathrm{FM}}(\omega_{\mathrm{d}},\omega_\tau)=\frac{1}{16}\frac{1}{(\omega_{\mathrm{d}}-2\omega_0)^2+4\gamma_0^2}\left\{\frac{4\gamma_0}{(\omega_\tau-\omega_0)^2+\gamma_0^2}+\frac{4\gamma_0}{(\omega_\tau-(\omega_{\mathrm{d}}-\omega_0))^2+\gamma_0^2}\right\}. \tag{12}$$

At the SH frequency ($\omega_{\mathrm{d}}=2\omega_0$), it reduces to

$$\hat{I}_{\mathrm{IF}}^{\mathrm{FM}}(\omega_{\mathrm{d}}=2\omega_0,\omega_\tau)=\frac{1}{8\gamma_0}\frac{1}{(\omega_\tau-\omega_0)^2+\gamma_0^2}. \tag{13}$$

This describes exactly the line shape of the fundamental Lorentzian oscillator which is resonant at ω_0 and has a dephasing rate of γ_0. This shows that the cross-sectional spectrum along the ω_τ axis gives the excitation profile of the fundamental resonance that generates the SH emission. This is seen in the numerical simulation, with a cross section of the FM band at $\omega_{\mathrm{d}}=2\omega_0$ that matches well to the linear spectrum of the Lorentzian (Supplementary Fig. 8d, top inset).

Above, we considered a δ-pulse excitation of the Lorentzian oscillator. In a realistic case of broadband, short pulse excitation, the linear convolution between the laser field and the response functions results in coherent oscillations of the local plasmonic field which, during the pulse reflect the instantaneous frequency of the driving laser. After the pulse, the field shows a free-induction decay with damped oscillations at the eigenfrequency $\omega_{\mathrm{S}}=\sqrt{\omega_0^2-\gamma_0^2}$ and with dephasing rate of γ_0, provided that the resonance is much narrower than the spectral width of laser. In this case, the IFRAC traces can be simulated by numerically simulating $E_{\mathrm{P}}(t)$ and inserting it into Eq. (2). Such a simulation, using the experimentally retrieved time structure $E_{\mathrm{L}}(t)$ of our laser pulses, is shown in Supplementary Fig. 9, taken the same parameters of the Lorentzian oscillator as in Supplementary Fig. 8. Since the linewidth of the plasmon resonance is much narrower than the laser spectrum, the resulting DC band is generally symmetric around ω_{d} and ω_τ and is only slightly narrower than expected from the purely Lorentzian response because of the convolution. Also, the cross-sectional spectrum of the FM band at the SH frequency matches quite well to that of the fundamental convoluted spectrum (Supplementary Fig. 9d). A closer examination, however, reveals some substructure of the FM band that reflects sum-frequency (SF) generation from different parts of the complex laser spectrum, as will be discussed in Supplementary Note 7.

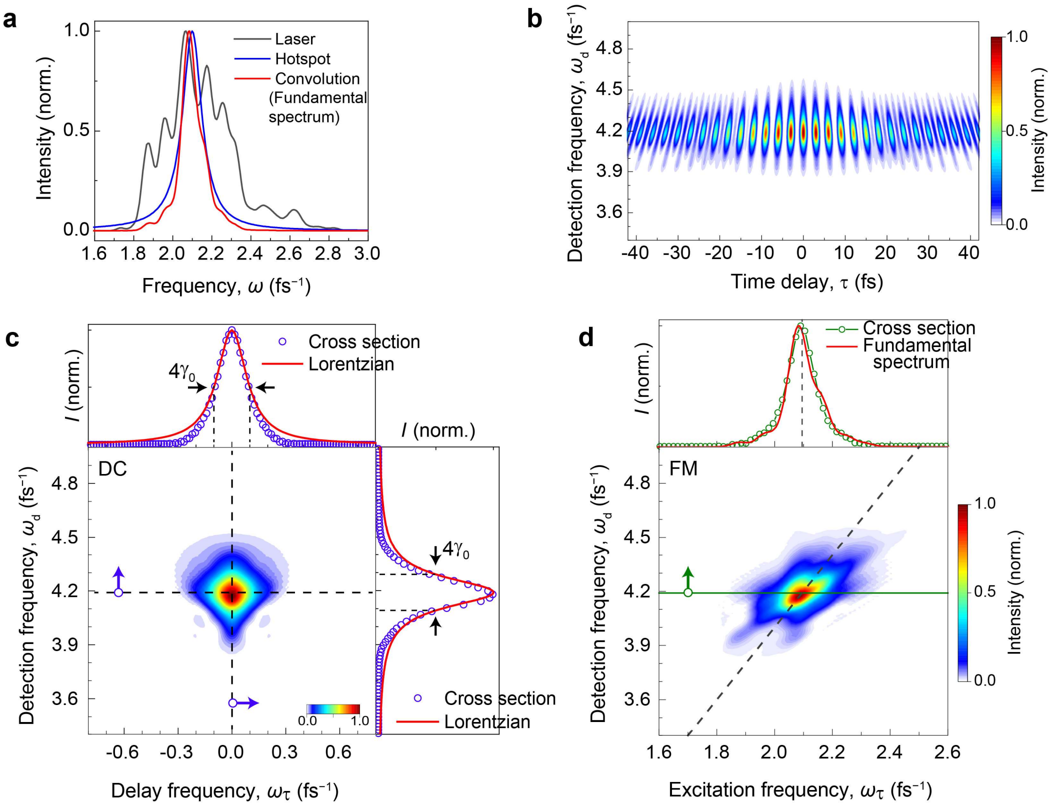

Supplementary Fig. 9 | Numerical simulation of the IFRAC trace of a single plasmon mode excited by an ultrashort laser field. (a) Linear spectrum of the experimentally used laser (gray curve), hot spot resonance (blue curve, a Lorentzian response function with ω_0 = 2.1 fs^{-1} and γ_0 = 0.06 fs^{-1}), and convoluted spectrum (red curve) after laser excitation. (b) Simulated IFRAC trace. (c) Fourier-transformed DC band, showing essentially a symmetric line shape along ω_d and ω_τ . Insets: Horizonal and vertical cross sections (blue circles) taken along the dashed lines, showing slightly reduced line widths compared to the Lorentzian response function in Eq. (1) due to the convolution with the finite-bandwidth laser pulses. (d) The FM band of the IFRAC trace, symmetric with respect to the diagonal line with a slope of 2. Top inset: cross-sectional spectrum (green circles) taken at the SH frequency of ω_d = 4.19 fs^{-1} as marked by the green line in the image. This spectrum matches the linear power spectrum of the local plasmon field $E_p(t)$ of the hot spot (red curve).

Supplementary Note 6: Inhomogeneous broadening of SH emission from multiple hot spots - coherent and incoherent model

The SH emission from our nanosponges is given by the superposition of hot spot fields from different, spatially highly localized plasmonic hot spots. A realistic modelling of the experimental results therefore needs to consider the SH emission from an inhomogeneously broadened ensemble of plasmonic hot spots. For this, we model each hot spot as a Lorentzian oscillator with response function $r_j(t) = \Theta(t)\sin(\omega_j t)e^{-\gamma_j t}$, where ω_j is the resonance frequency, γ_j the dephasing rate, and j the hot spot index. Since the hot spots are spatially localized to a 10-nm scale[13] and are spatially well-separated without appreciable overlap of the plasmon modes, each hot spot is treated as an independent nonlinear emitter, generating a local nonlinear field $E_{NL}^{j}(t,\tau)$, as confirmed by tr-PEEM measurements. To illustrate the difference between coherent and incoherent sum of the SH emission from the ensemble of hot spots, we consider an

ensemble of 20 hot spots, centred at $\omega_0 = 2.25$ fs^{-1} with an equidistant frequency spacing $\Delta\omega = 0.06$ fs^{-1}. Their amplitudes are defined by a Lorentzian distribution function in that frequency range. The dephasing rates of all hot spots are set to be the same, $\gamma_j = 0.06$ fs^{-1}, determined from the fitting of the DC band of the experimental data (Fig. 1c in main text). The response functions in the frequency domain $\tilde{r}_j(\omega) = \int_0^\infty r_j(t) e^{i\omega t} dt$ are plotted in Supplementary Fig. 10a. The local surface plasmon fields of hot spots are given by the convolution of the response with the external laser field. Here, a Gaussian-shaped laser with ~7-fs pulse duration is used as the excitation source to avoid the multi-peaked structure of the experimentally used laser source (Supplementary Fig. 10b).

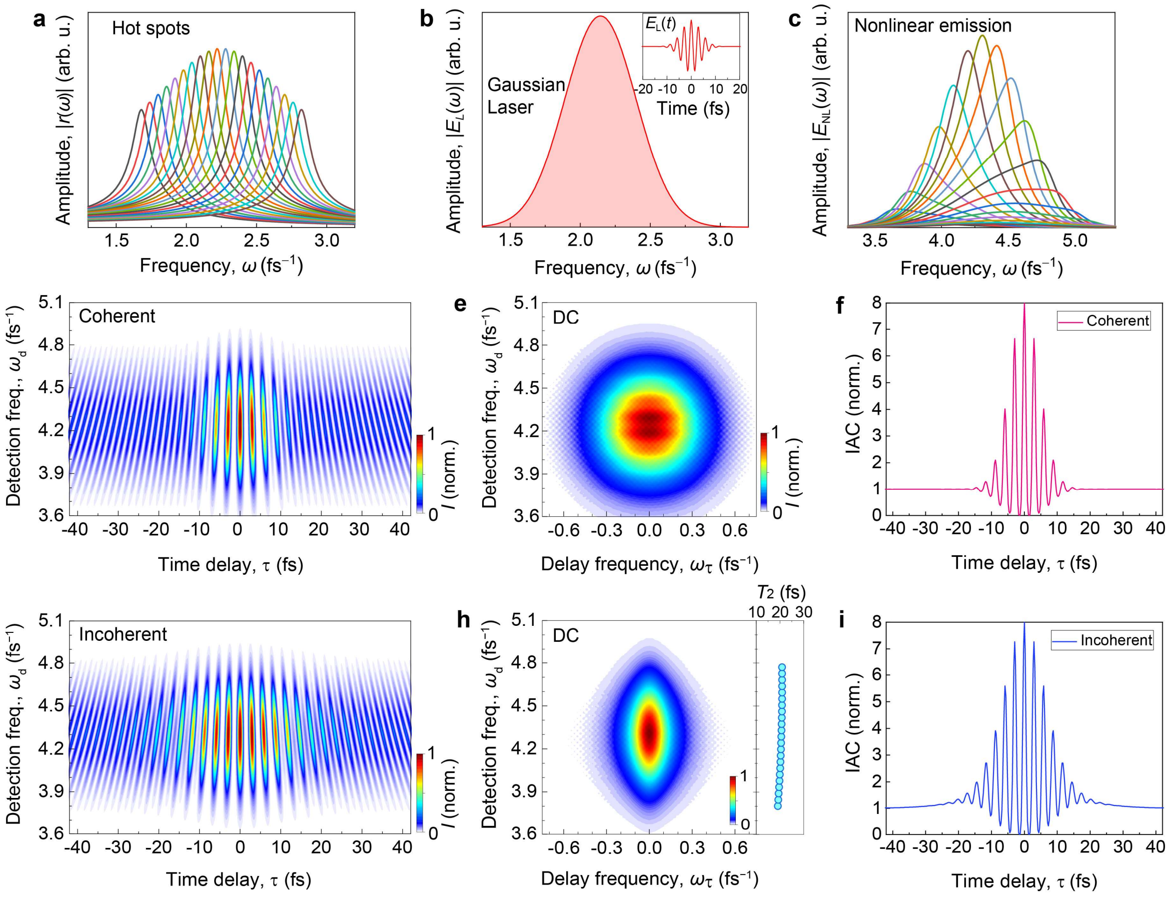

Supplementary Fig. 10 | Simulation of 2nd-order nonlinear emission from multiple hot spots–a comparison of coherent and incoherent emission models. (a) Field amplitudes of response functions of 20 evenly spaced Lorentzian oscillators with a frequency spacing $\Delta\omega = 0.06$ fs^{-1} taken to represent an ensemble of plasmonic hot spot modes. All modes have a same dephasing rate of $\gamma_j = 0.06$ fs^{-1}. (b) Field amplitude and time profile (inset) of a Gaussian-shaped excitation laser. (c) Amplitudes of SH fields of the 20 hot spots at $\tau = 0$, considering each hot spot as an independent nonlinear emitter. (d) IFRAC trace simulated in the coherent model by coherently interfering the SH fields from all hot spots. (e) Fourier-transformed DC band and (f) IAC trace of the IFRAC in (d). (g) IFRAC trace simulated in the incoherent model by adding the intensities of the SH emission from all hot spots. (h) Fourier-transformed DC band and (i) IAC trace of the IFRAC in (g). The inset in (h) shows the extracted dephasing time $T_{2,j} \sim 20$ fs ($\gamma_j \sim 0.05$ fs^{-1}) obtained by Lorentzian fits at the different ω_d.

In a coherent model, IFRAC is taken by coherently adding up the nonlinear fields emitted from all hot spots $I'_{\mathrm{IF}}(\omega_{\mathrm{d}},\tau)=\left|\sum_j \tilde{E}^j_{\mathrm{NL}}(\omega_{\mathrm{d}},\tau)\right|^2$. In an incoherent model, IFRAC is calculated as the incoherent sum $I'_{\mathrm{IF}}(\omega_{\mathrm{d}},\tau)=\sum_j \left|\tilde{E}^j_{\mathrm{NL}}(\omega_{\mathrm{d}},\tau)\right|^2$. The latter supresses the interference between emitted nonlinear fields from different hot spots.[14] We emphasize that, even in the discussed incoherent model, the SH emission from each hot spot is still a coherent process. The simulated IFRAC, DC band, and IAC traces are shown in Supplementary Fig. 10. The coherent model (Supplementary Fig. 10 d-f) shows a circularly-shaped DC band, with a linewidth along the ω_τ axis which is given by the inhomogeneous linewidth of the ensemble of plasmonic hotsports.[15] In such coherent emission scenario, the IFRAC trace, based on a 2nd-order nonlinear optical measurement, cannot distinguish between homogeneous and inhomogeneous broadening.[15] This result is evidently in stark contrast with the experimental result in Fig. 1c of the main manuscript. Furthermore, the IAC trace of the coherent model displays short-lived coherent oscillations, decaying on a time scale which is again given by the inhomogeneous linewidth. This is again very different from the experimental result.

Instead, the DC band of the incoherent model is clearly stretched in ω_{d} axis (Supplementary Fig. 10h), very similar to our experimental observation in Fig. 1c. In such an incoherent emission case, the linewidth along the ω_τ axis is given by that of the homogeneously broadened single oscillator resonance. A fitting of the cross section of DC band at different ω_{d} gives $\gamma\sim$ 0.05 fs^{-1}, corresponding to T_2 time of ~ 20 fs. This is very close to the originally set value of γ_j = 0.06 fs^{-1}. The slight reduction of γ results from the linear convolution between laser field and response function. Also, the simulated IAC trace shows much longer-lived coherent oscillations, decaying on a time scale given by the single-oscillator dephasing time $T_2=1/\gamma$. The IAC curves also display a distinct up-tilted wing structure (for $\tau=\pm10$–30 fs), which is also observed in the experimental data (see data of bare gold nanosponge in Supplementary Fig. 13c and Au/ZnO hybrid nanosponge in Fig. 3c in the main text).

Indeed, as discussed in the main text, the SH fields from randomly disordered (percolated) metal thin films have been predicted to show SH emission from localized hot spots with negligible interference between the fields from neighbouring hot spots.[14] Experimentally, this has mainly been confirmed by studying the polarization properties of SH from such disordered films.[16] Such experiments suggest a complex, multipolar character of the emission with strong variations on small length scales[16, 17] similar to that seen in Fig. 1f. This may be taken as an additional support for assuming incoherent emission model to simulate the SH from our sample.

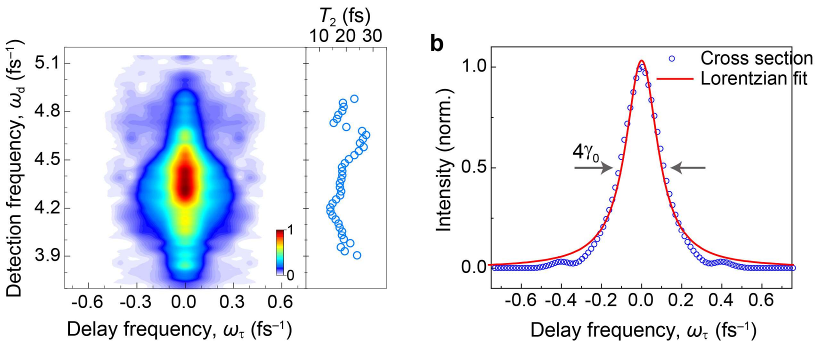

Supplementary Fig. 11 | Lorentzian fitting of the DC band to estimate the homogeneously broadened linewidth of plasmonic hot spots. (a) Fourier-transformed DC band of the IFRAC trace and the fitted homogeneous dephasing time as a function of ω_d. The data are the same as that in Fig. 1c of the main text. (b) Representative horizontal cross section of the DC band at $\omega_d = 4.5$ fs^{-1} (blue circles), fitted to a Lorentzian response (Eq. (8)) with linewidth $4\gamma_0$. We extract $\gamma_0 \sim 0.06$ fs^{-1}, indicating a dephasing time of $T_2 \sim 16$ fs for a single hot spot.

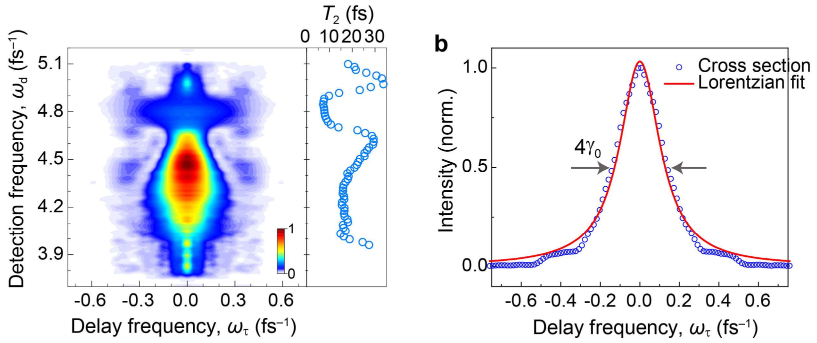

Supplementary Fig. 12 | Lorentzian fitting of the DC band of the Au/ZnO hybrid nanosponge. (a) Fourier-transformed DC band of the IFRAC trace of the hybrid nanosponge shown in Fig. 3b. Right inset: fitted homogeneous dephasing time as a function of ω_d. (b) Representative horizontal cross section of the DC band at $\omega_d = 4.2$ fs^{-1} (blue circles), fitted to a Lorentzian response (Eq. (8)) with linewidth $4\gamma_0$. The fitted $\gamma_0 \sim 0.06$ fs^{-1} and $T_2 = 1/\gamma_0$ of ~ 16 fs is close to the values of the bare gold nanosponge. Interestingly, a pronounced increase in linewidth occurs around $\omega_d = \omega_X \sim 4.8$ fs^{-1}, i.e., near the ZnO bandgap. This may be a signature of nonlinear plasmon-exciton coupling, specifically plasmon-enhanced sum-frequency generation from ZnO excitons.

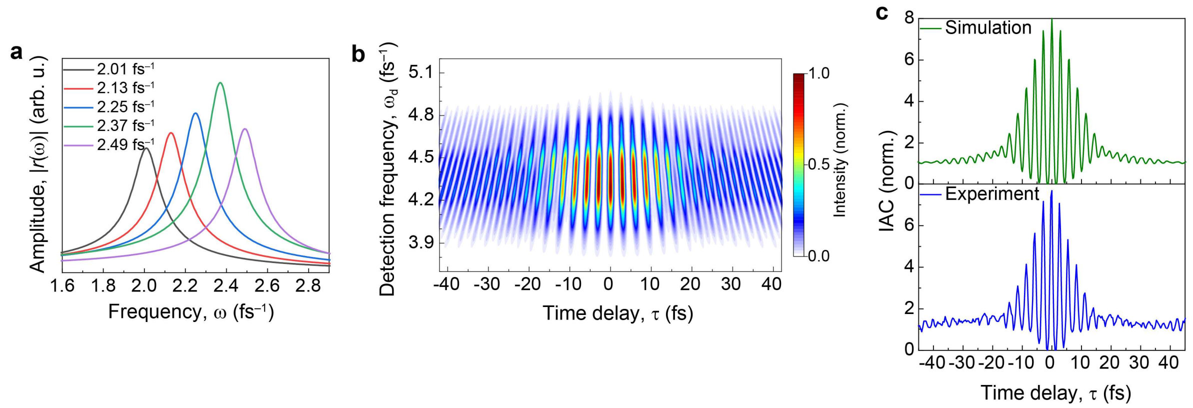

Supplementary Fig. 13 | Modelling of the IFRAC trace of the bare gold nanosponge in Fig. 1b. (a) Field amplitudes of the response functions of 5 evenly spaced Lorentzian oscillators, modelling the plasmonic hot spot response, centred at ω_0 = 2.25 fs^{-1} with a frequency spacing $\Delta\omega$ = 0.12 fs^{-1}. (b) Simulated IFRAC trace from the 5 hot spots excited by the experimental laser field, using the incoherent SH model discussed above. The Fourier-transformed DC band of this IFRAC trace is shown in Fig. 1d in the main text. (c) Interferometric autocorrelation (IAC) traces of the simulated IFRAC (top), showing reasonable agreement with the experimental data integrated from the IFRAC trace in Fig. 1b (bottom).

The analysis of DC band in the simulation shown in Supplementary Fig. 10 suggests that a fitting of the horizontal cross sections of DC band at different ω_d provides the homogeneous dephasing rate of hot spots. We have applied this fitting to the experimental data of the bare gold (Supplementary Fig. 11) and hybrid Au/ZnO nanosponges (Supplementary Fig. 12) and obtained an averaged dephasing time T_2 ~ 16 fs for the hot spots. Using the deduced T_2 time and the incoherent SH emission model, we have simulated IFRAC trace of the bare gold nanosponge, assuming SH emission from an ensemble of 5 hot spots (Supplementary Fig. 13a). The simulated IFRAC trace is shown in Supplementary Fig. 13b. It matches reasonably well to the experimental data in Fig. 1b of the main manuscript. The simulated Fourier-transformed DC band (Fig. 1d of the main text) and IAC trace (Supplementary Fig. 13c) also agrees reasonably well with the experiment. We thus conclude that the nonlinear plasmonic emission from gold nanosponges is showing the inhomogeneously broadened SH emission from several spectrally narrow and long-lived hot spot modes.

Supplementary Note 7: Analytical model for IFRAC of two coherently superposed modes

Time-domain IFRAC trace

The IAC trace near the excitonic emission peak of the hybrid Au/ZnO nanosponge in Fig. 3c shows a clear beating pattern, which necessarily implies that a coherent superposition of at least two modes contributes to the signal. We now examine the effect of a coherent superposition of two modes on the IFRAC signal. Note that the two 'modes' discussed do not necessarily need to be two distinct eigenmodes of the material system under investigation. Due to the broad linewidth of the laser pulses, they could also from the coupling of the sub-structured laser field to a single resonance. Specifically, two frequency components in the laser field, naturally containing multiple modes, give rise to two frequency components in the plasmonic or ZnO field after the convolution with laser, provided that these two frequency components are phase locked. For analytical purposes, we model the two modes as two

Lorentzian oscillators with angular frequencies ω_1 and ω_2, and dephasing rates γ_1 and γ_2, respectively.

The response function of the two coherent superposed modes, $r(t)=\Theta(t)(\sin(\omega_1 t)e^{-\gamma_1 t}+\sin(\omega_2 t)e^{-\gamma_2 t})$, leads to a beating in the time domain (Supplementary Fig. 14a) and gives a response function in the frequency domain containing two peaks,

$$\tilde{r}(\omega)=\int_0^{\infty} r(t)e^{i\omega t}dt=\frac{-1}{2}\left(\frac{1}{\omega-\omega_1+i\gamma_1}-\frac{1}{\omega+\omega_1+i\gamma_1}+\frac{1}{\omega-\omega_2+i\gamma_2}-\frac{1}{\omega+\omega_2+i\gamma_2}\right). \quad (14)$$

Again, to obtain analytical expressions, we assume an excitation with δ pulses. The 2nd-order nonlinear field is ($\tau>0$)

$$\begin{aligned}E_{NL}(t,\tau)&=(r(t)+r(t-\tau))^2\\&=\left\{\Theta(t)(\sin(\omega_1 t)e^{-\gamma_1 t}+\sin(\omega_2 t)e^{-\gamma_2 t})+\Theta(t-\tau)(\sin(\omega_1(t-\tau))e^{-\gamma_1(t-\tau)}+\sin(\omega_2(t-\tau))e^{-\gamma_2(t-\tau)})\right\}^2\end{aligned}. \quad (15)$$

After expansion and Fourier transform, we get the signal in frequency domain

$$\tilde{E}_{NL}(\omega_d,\tau)=-\frac{i}{4}\left\{\begin{array}{l}+\dfrac{1+e^{i\omega_d\tau}+2e^{i(\omega_d+\omega_1)\tau}e^{-\gamma_1\tau}}{\omega_d+2\omega_1+i2\gamma_1}+\dfrac{1+e^{i\omega_d\tau}+2e^{i(\omega_d-\omega_1)\tau}e^{-\gamma_1\tau}}{\omega_d-2\omega_1+i2\gamma_1}-\dfrac{2+2e^{i\omega_d\tau}+2e^{i\omega_d\tau}e^{-\gamma_1\tau}(2\cos(\omega_1\tau))}{\omega_d+i2\gamma_1}\\+\dfrac{1+e^{i\omega_d\tau}+2e^{i(\omega_d+\omega_2)\tau}e^{-\gamma_2\tau}}{\omega_d+2\omega_2+i2\gamma_2}+\dfrac{1+e^{i\omega_d\tau}+2e^{i(\omega_d-\omega_2)\tau}e^{-\gamma_2\tau}}{\omega_d-2\omega_2+i2\gamma_2}-\dfrac{2+2e^{i\omega_d\tau}+2e^{i\omega_d\tau}e^{-\gamma_2\tau}(2\cos(\omega_2\tau))}{\omega_d+i2\gamma_2}\\+\dfrac{2+2e^{i\omega_d\tau}+2e^{i(\omega_d+\omega_1)\tau}e^{-\gamma_1\tau}+2e^{i(\omega_d+\omega_2)\tau}e^{-\gamma_2\tau}}{\omega_d+(\omega_1+\omega_2)+i(\gamma_1+\gamma_2)}+\dfrac{2+2e^{i\omega_d\tau}+2e^{i(\omega_d-\omega_1)\tau}e^{-\gamma_1\tau}+2e^{i(\omega_d-\omega_2)\tau}e^{-\gamma_2\tau}}{\omega_d-(\omega_1+\omega_2)+i(\gamma_1+\gamma_2)}\\-\dfrac{2+2e^{i\omega_d\tau}+2e^{i(\omega_d+\omega_1)\tau}e^{-\gamma_1\tau}+2e^{i(\omega_d-\omega_2)\tau}e^{-\gamma_2\tau}}{\omega_d+(\omega_1-\omega_2)+i(\gamma_1+\gamma_2)}-\dfrac{2+2e^{i\omega_d\tau}+2e^{i(\omega_d-\omega_1)\tau}e^{-\gamma_1\tau}+2e^{i(\omega_d+\omega_2)\tau}e^{-\gamma_2\tau}}{\omega_d-(\omega_1-\omega_2)+i(\gamma_1+\gamma_2)}\end{array}\right\}.$$

(16)

We find that the first 6 terms are the same as the nonlinear generation of the single Lorentzian oscillator discussed in Supplementary Note 5, that is, the SH and OR signals of each of the two oscillators. Additionally, the last 4 terms reflect sum frequency (SF) and difference frequency generation of the two oscillators. In experiment, only the SH and SF in the positive frequency range are detected, and the frequency of the difference frequency signal is also too small and filtered, leaving

$$\tilde{E}_{NL}(\omega_d,\tau)=-\frac{i}{4}\left\{\begin{array}{l}\dfrac{1+e^{i\omega_d\tau}+2e^{i(\omega_d-\omega_1)\tau}e^{-\gamma_1\tau}}{\omega_d-2\omega_1+i2\gamma_1}+\\\dfrac{1+e^{i\omega_d\tau}+2e^{i(\omega_d-\omega_2)\tau}e^{-\gamma_2\tau}}{\omega_d-2\omega_2+i2\gamma_2}+\\\dfrac{2+2e^{i\omega_d\tau}+2e^{i(\omega_d-\omega_1)\tau}e^{-\gamma_1\tau}+2e^{i(\omega_d-\omega_2)\tau}e^{-\gamma_2\tau}}{\omega_d-(\omega_1+\omega_2)+i(\gamma_1+\gamma_2)}\end{array}\right\}. \quad (17)$$

The IFRAC intensity $I_{IF}(\omega_d,\tau)=|E_{NL}(\omega_d,\tau)|^2$ contains 6 terms (neglecting the constant factor of 1/16),

$$I_{\mathrm{IF}}^{\mathrm{SH1}}(\omega_{\mathrm{d}},\tau)=\frac{1}{(\omega_{\mathrm{d}}-2\omega_1)^2+4\gamma_1^2}\left\{2+4\mathrm{e}^{-2\gamma_1\tau}+2\cos(\omega_{\mathrm{d}}\tau)+4\cos(\omega_1\tau)\mathrm{e}^{-\gamma_1\tau}+4\cos((\omega_{\mathrm{d}}-\omega_1)\tau)\mathrm{e}^{-\gamma_1\tau}\right\} \tag{18}$$

$$I_{\mathrm{IF}}^{\mathrm{SH2}}(\omega_{\mathrm{d}},\tau)=\frac{1}{(\omega_{\mathrm{d}}-2\omega_2)^2+4\gamma_2^2}\left\{2+4\mathrm{e}^{-2\gamma_2\tau}+2\cos(\omega_{\mathrm{d}}\tau)+4\cos(\omega_2\tau)\mathrm{e}^{-\gamma_2\tau}+4\cos((\omega_{\mathrm{d}}-\omega_2)\tau)\mathrm{e}^{-\gamma_2\tau}\right\} \tag{19}$$

$$I_{\mathrm{IF}}^{\mathrm{SF}}(\omega_{\mathrm{d}},\tau)=\frac{4}{\left(\omega_{\mathrm{d}}-(\omega_1+\omega_2)\right)^2+(\gamma_1+\gamma_2)^2}\left\{\begin{array}{l}2+\mathrm{e}^{-2\gamma_1\tau}+\mathrm{e}^{-2\gamma_2\tau}+2\cos(\omega_{\mathrm{d}}\tau)+2\cos(\omega_1\tau)\mathrm{e}^{-\gamma_1\tau}+2\cos(\omega_2\tau)\mathrm{e}^{-\gamma_2\tau}+\\2\cos\left((\omega_{\mathrm{d}}-\omega_1)\tau\right)\mathrm{e}^{-\gamma_1\tau}+2\cos\left((\omega_{\mathrm{d}}-\omega_2)\tau\right)\mathrm{e}^{-\gamma_2\tau}+2\cos\left((\omega_1-\omega_2)\tau\right)\mathrm{e}^{-(\gamma_1+\gamma_2)\tau}\end{array}\right\}$$

(20

$$I_{\mathrm{IF}}^{\mathrm{interfere}}(\omega_{\mathrm{d}},\tau)=$$

$$\frac{2}{a^2+b^2}\left\{\begin{array}{l}a\left[\begin{array}{l}2+2\cos(\omega_{\mathrm{d}}\tau)+2\cos(\omega_1\tau)\mathrm{e}^{-\gamma_1\tau}+2\cos(\omega_2\tau)\mathrm{e}^{-\gamma_2\tau}+\\2\cos((\omega_{\mathrm{d}}-\omega_1)\tau)\mathrm{e}^{-\gamma_1\tau}+2\cos((\omega_{\mathrm{d}}-\omega_2)\tau)\mathrm{e}^{-\gamma_2\tau}+4\cos((\omega_1-\omega_2)\tau)\mathrm{e}^{-(\gamma_1+\gamma_2)\tau}\end{array}\right]+\\b\left[\begin{array}{l}-2\sin(\omega_1\tau)\mathrm{e}^{-\gamma_1\tau}+2\sin(\omega_2\tau)\mathrm{e}^{-\gamma_2\tau}\\+2\sin((\omega_{\mathrm{d}}-\omega_1)\tau)\mathrm{e}^{-\gamma_1\tau}-2\sin((\omega_{\mathrm{d}}-\omega_2)\tau)\mathrm{e}^{-\gamma_2\tau}-4\sin((\omega_1-\omega_2)\tau)\mathrm{e}^{-(\gamma_1+\gamma_2)\tau}\end{array}\right]\end{array}\right\}+$$

$$\frac{2}{c^2+d^2}\left\{\begin{array}{l}c\left\{\begin{array}{l}4+4\mathrm{e}^{-2\gamma_1\tau}+4\cos(\omega_{\mathrm{d}}\tau)+6\cos(\omega_1\tau)\mathrm{e}^{-\gamma_1\tau}+2\cos(\omega_2\tau)\mathrm{e}^{-\gamma_2\tau}\\+6\cos((\omega_{\mathrm{d}}-\omega_1)\tau)\mathrm{e}^{-\gamma_1\tau}+2\cos((\omega_{\mathrm{d}}-\omega_2)\tau)\mathrm{e}^{-\gamma_2\tau}+4\cos((\omega_1-\omega_2)\tau)\mathrm{e}^{-(\gamma_1+\gamma_2)\tau}\end{array}\right\}+\\d\left\{\begin{array}{l}-2\sin(\omega_1\tau)\mathrm{e}^{-\gamma_1\tau}+2\sin(\omega_2\tau)\mathrm{e}^{-\gamma_2\tau}\\+2\sin((\omega_{\mathrm{d}}-\omega_1)\tau)\mathrm{e}^{-\gamma_1\tau}-2\sin((\omega_{\mathrm{d}}-\omega_2)\tau)\mathrm{e}^{-\gamma_2\tau}-4\sin((\omega_1-\omega_2)\tau)\mathrm{e}^{-(\gamma_1+\gamma_2)\tau}\end{array}\right\}\end{array}\right\}+ \tag{21}$$

$$\frac{2}{g^2+h^2}\left\{\begin{array}{l}g\left\{\begin{array}{l}4+4\mathrm{e}^{-2\gamma_2\tau}+4\cos(\omega_{\mathrm{d}}\tau)+2\cos(\omega_1\tau)\mathrm{e}^{-\gamma_1\tau}+6\cos(\omega_2\tau)\mathrm{e}^{-\gamma_2\tau}\\+2\cos((\omega_{\mathrm{d}}-\omega_1)\tau)\mathrm{e}^{-\gamma_1\tau}+6\cos((\omega_{\mathrm{d}}-\omega_2)\tau)\mathrm{e}^{-\gamma_2\tau}+4\cos((\omega_1-\omega_2)\tau)\mathrm{e}^{-(\gamma_1+\gamma_2)\tau}\end{array}\right\}+\\h\left\{\begin{array}{l}+2\sin(\omega_1\tau)\mathrm{e}^{-\gamma_1\tau}-2\sin(\omega_2\tau)\mathrm{e}^{-\gamma_2\tau}\\-2\sin((\omega_{\mathrm{d}}-\omega_1)\tau)\mathrm{e}^{-\gamma_1\tau}+2\sin((\omega_{\mathrm{d}}-\omega_2)\tau)\mathrm{e}^{-\gamma_2\tau}+4\sin((\omega_1-\omega_2)\tau)\mathrm{e}^{-(\gamma_1+\gamma_2)\tau}\end{array}\right\}\end{array}\right\}$$

with

$$a=(\omega_{\mathrm{d}}-2\omega_1)(\omega_{\mathrm{d}}-2\omega_2)+4\gamma_1\gamma_2,\ b=2\gamma_1(\omega_{\mathrm{d}}-2\omega_2)-2\gamma_2(\omega_{\mathrm{d}}-2\omega_1),$$

$$c=(\omega_{\mathrm{d}}-2\omega_1)\left(\omega_{\mathrm{d}}-(\omega_1+\omega_2)\right)+2\gamma_1(\gamma_1+\gamma_2)\ ,\ d=2\gamma_1\left(\omega_{\mathrm{d}}-(\omega_1+\omega_2)\right)-(\gamma_1+\gamma_2)(\omega_{\mathrm{d}}-2\omega_1),$$

$$g=(\omega_{\mathrm{d}}-2\omega_2)\left(\omega_{\mathrm{d}}-(\omega_1+\omega_2)\right)+2\gamma_2(\gamma_1+\gamma_2),\ h=2\gamma_2\left(\omega_{\mathrm{d}}-(\omega_1+\omega_2)\right)-(\gamma_1+\gamma_2)(\omega_{\mathrm{d}}-2\omega_2).$$

The formula for the intensity of the time-dependent IFRAC trace contains two SH terms (Eqs. (18) and (19)), one SF term (Eq. (20)), and three interference terms between them (Eq. (21)), which define the line shape of the emission spectra along ω_{d} axis. The signal is again modulated as a function of τ. A simulated IFRAC trace is presented in Supplementary Fig. 14b.

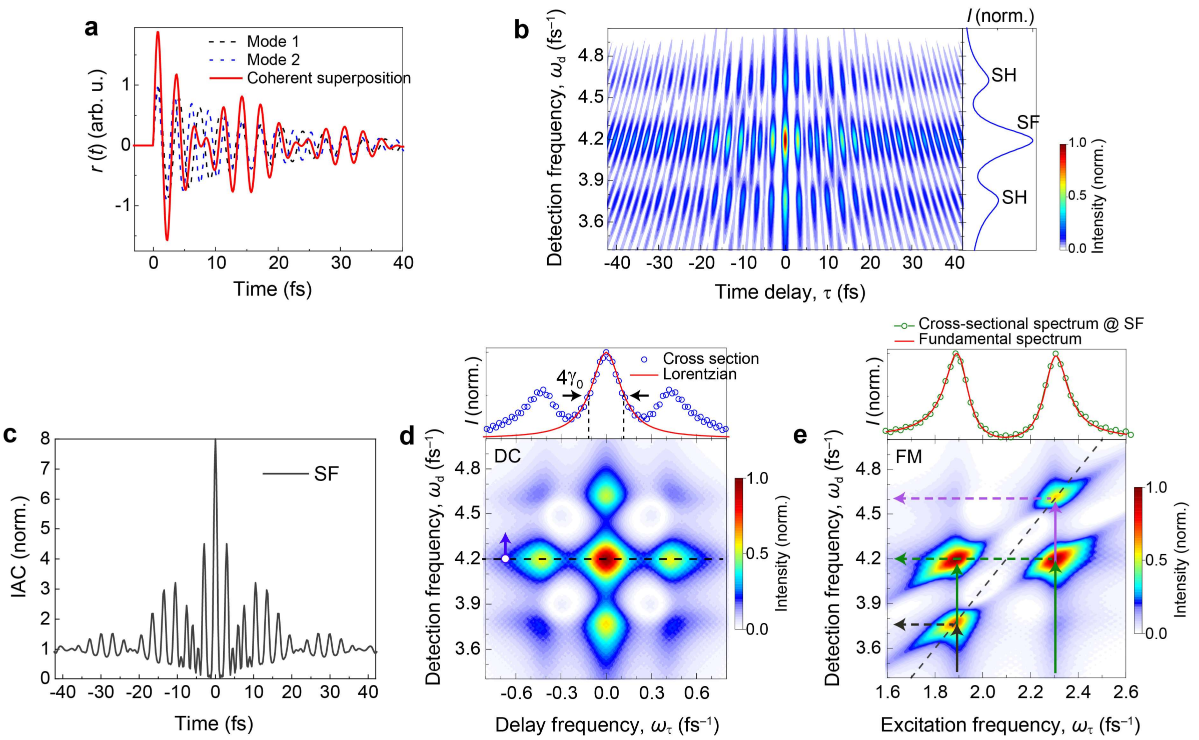

Supplementary Fig. 14 | Simulated IFRAC trace from two coherently superposed modes. (a) Time-domain response function $r(t)$ of the two modes, represented as Lorentzian oscillators, and their coherent superposition, which shows a clear beating pattern with a period $T = 2\pi/(\omega_2 - \omega_1) \sim 15.7$ fs. Parameters of the two modes: $\omega_1 = 1.9\ \mathrm{fs}^{-1}$, $\omega_2 = 2.3\ \mathrm{fs}^{-1}$, $\gamma_1 = \gamma_2 = 0.06\ \mathrm{fs}^{-1}$, the amplitudes are the same. (b) Simulated IFRAC trace of the coherently superposed modes. Right inset: nonlinear emission spectrum showing not only two SH peaks at $\omega_\mathrm{d} \sim 3.8$ and $4.6\ \mathrm{fs}^{-1}$ but also a SF peak at $\omega_\mathrm{d} \sim 4.2\ \mathrm{fs}^{-1}$. (c) Interferometric autocorrelation (IAC) trace of the SF peak integrated between ω_d = 3.95–4.45 fs^{-1}, showing the beating pattern. (d) Fourier-transformed DC band of the IFRAC trace. Top inset: horizontal cross section (blue circles) taken at SF peak ($\omega_\mathrm{d} \sim 4.2\ \mathrm{fs}^{-1}$) marked by the dashed line in the image. The red curve plots the line shape of the Lorentzian function with a width of $4\gamma_0$ ($\gamma_0 = \gamma_1 = \gamma_2$) described in Eq. (24). The sidebands at $\omega_\tau \sim \pm 0.4\ \mathrm{fs}^{-1}$ in the SF peak ($\omega_\mathrm{d} \sim 4.2\ \mathrm{fs}^{-1}$) appear at the difference frequency of the two modes (Eq. (28)). (e) FM band of the IFRAC trace, correlating fundamental excitation frequencies to 2nd-order emission frequencies as marked by arrows. The splitting at $\omega_\mathrm{d} = 4.2\ \mathrm{fs}^{-1}$, symmetric with respect to the diagonal dashed line with a slope of 2 indicates a SF signal, whereas the signal centered at the diagonal line indicates the SH signal. Top inset: cross-sectional spectrum (green circles) taken at the SF peak ($\omega_\mathrm{d} = 4.2\ \mathrm{fs}^{-1}$) from the FM band, which matches to the linear spectrum of the coherently superposed modes (red curve) according to Eq. (26).

Fourier transform along the time delay axis

The two SH terms have exactly the same form as that of the single Lorentzian oscillator, and thus their Fourier transform and the analysis are the same. We are most interested in the Fourier transform of the SF term,

$$\tilde{I}_{\mathrm{IF}}^{\mathrm{SF}}(\omega_\mathrm{d}, \omega_\tau) = \int_{-\infty}^{\infty} I_{\mathrm{IF}}^{\mathrm{SF}}(\omega_\mathrm{d}, \tau) \mathrm{e}^{\mathrm{i}\omega_\tau \tau} d\tau = \frac{4}{(\omega_\mathrm{d} - (\omega_1 + \omega_2))^2 + (\gamma_1 + \gamma_2)^2} \hat{I}_{\mathrm{IF}}^{\mathrm{SF}}(\omega_\mathrm{d}, \omega_\tau), \tag{22}$$

with

$$\tilde{I}_{\mathrm{IF}}^{\mathrm{SF}}(\omega_{\mathrm{d}},\omega_\tau)=\begin{Bmatrix} 4\pi\delta(\omega_\tau)+\frac{4\gamma_1}{\omega_\tau^2+4\gamma_1^2}+\frac{4\gamma_2}{\omega_\tau^2+4\gamma_2^2}+2\pi\delta(\omega_\tau+\omega_{\mathrm{d}})+2\pi\delta(\omega_\tau-\omega_{\mathrm{d}})+ \\ \frac{2\gamma_1}{(\omega_\tau+\omega_1)^2+\gamma_1^2}+\frac{2\gamma_1}{(\omega_\tau-\omega_1)^2+\gamma_1^2}+\frac{2\gamma_2}{(\omega_\tau+\omega_2)^2+\gamma_2^2}+\frac{2\gamma_2}{(\omega_\tau-\omega_2)^2+\gamma_2^2}+ \\ \frac{2\gamma_1}{(\omega_\tau+(\omega_{\mathrm{d}}-\omega_1))^2+\gamma_1^2}+\frac{2\gamma_1}{(\omega_\tau-(\omega_{\mathrm{d}}-\omega_1))^2+\gamma_1^2}+\frac{2\gamma_2}{(\omega_\tau+(\omega_{\mathrm{d}}-\omega_2))^2+\gamma_2^2}+\frac{2\gamma_2}{(\omega_\tau-(\omega_{\mathrm{d}}-\omega_2))^2+\gamma_2^2} \\ \frac{2(\gamma_1+\gamma_2)}{(\omega_\tau+(\omega_1-\omega_2))^2+(\gamma_1+\gamma_2)^2}+\frac{2(\gamma_1+\gamma_2)}{(\omega_\tau-(\omega_1-\omega_2))^2+(\gamma_1+\gamma_2)^2} \end{Bmatrix}. \tag{23}$$

We skip the delta function terms in the DC and the 2nd-order bands and discuss only the relevant terms containing the information about the studied system. The DC band,

$$\tilde{I}_{\mathrm{IF}}^{\mathrm{SF,DC}}(\omega_{\mathrm{d}},\omega_\tau)=\frac{4}{(\omega_{\mathrm{d}}-(\omega_1+\omega_2))^2+(\gamma_1+\gamma_2)^2}\left\{\frac{4\gamma_1}{\omega_\tau^2+4\gamma_1^2}+\frac{4\gamma_2}{\omega_\tau^2+4\gamma_2^2}\right\} \tag{24}$$

again shows a symmetric 2D line shape along ω_{d} and ω_τ. This is seen in the numerical simulation in Supplementary Fig. 14d.

The FM band, limited to positive ω_τ, reads

$$\tilde{I}_{\mathrm{IF}}^{\mathrm{SF,FM}}(\omega_{\mathrm{d}},\omega_\tau)=\frac{4}{(\omega_{\mathrm{d}}-(\omega_1+\omega_2))^2+(\gamma_1+\gamma_2)^2}\begin{Bmatrix} \frac{2\gamma_1}{(\omega_\tau-\omega_1)^2+\gamma_1^2}+\frac{2\gamma_2}{(\omega_\tau-\omega_2)^2+\gamma_2^2}+ \\ \frac{2\gamma_1}{(\omega_\tau-(\omega_{\mathrm{d}}-\omega_1))^2+\gamma_1^2}+\frac{2\gamma_2}{(\omega_\tau-(\omega_{\mathrm{d}}-\omega_2))^2+\gamma_2^2} \end{Bmatrix}. \tag{25}$$

The cross-sectional spectrum at the SF peak at $\omega_{\mathrm{d}} = \omega_1 + \omega_2$ becomes

$$\tilde{I}_{\mathrm{IF}}^{\mathrm{SF,FM}}(\omega_{\mathrm{d}}=\omega_1+\omega_2,\omega_\tau)=\frac{4}{(\gamma_1+\gamma_2)^2}\left\{\frac{2\gamma_1}{(\omega_\tau-\omega_1)^2+\gamma_1^2}+\frac{2\gamma_2}{(\omega_\tau-\omega_1)^2+\gamma_2^2}+\frac{2\gamma_2}{(\omega_\tau-\omega_2)^2+\gamma_2^2}+\frac{2\gamma_1}{(\omega_\tau-\omega_2)^2+\gamma_1^2}\right\}. \tag{26}$$

If the dephasing rates of the two modes are comparable, $\gamma = \gamma_1 \approx \gamma_2$, it reduces to

$$\tilde{I}_{\mathrm{IF}}^{\mathrm{SF,FM}}(\omega_{\mathrm{d}}=\omega_1+\omega_2,\omega_\tau)=\frac{4}{\gamma}\left\{\frac{1}{(\omega_\tau-\omega_1)^2+\gamma^2}+\frac{1}{(\omega_\tau-\omega_2)^2+\gamma^2}\right\}. \tag{27}$$

The cross-sectional spectrum at the SF peak is composed of the linear spectrum of the two fundamental Lorentzian oscillators that are mixed to generate the SF signal. This is observed in the numerical simulation result in Supplementary Fig. 14e and in the experimental data recorded using a BBO crystal (Supplementary Fig. 15). Therefore, taken together with the analytical result of the single oscillator (Eq. (12)), we conclude that the cross-sectional spectrum taken at a specific ω_{d} in the FM band, shows the linear spectrum of the fundamental modes that are mixed to generate the nonlinear signal at that ω_{d}. This provides the analytical basis for correlating linear excitation and 2nd-order nonlinear emission frequencies in the FM band signal.

Additionally, there is another band,

$$\tilde{I}_{\mathrm{IF}}^{\mathrm{SF,DC'}}(\omega_{\mathrm{d}},\omega_\tau)=\frac{4}{(\omega_{\mathrm{d}}-(\omega_1+\omega_2))^2+(\gamma_1+\gamma_2)^2}\left\{\frac{2(\gamma_1+\gamma_2)}{(\omega_\tau+(\omega_1-\omega_2))^2+(\gamma_1+\gamma_2)^2}+\frac{2(\gamma_1+\gamma_2)}{(\omega_\tau-(\omega_1-\omega_2))^2+(\gamma_1+\gamma_2)^2}\right\} \tag{28}$$

that shows a resonance peak at the difference frequency $\omega_\tau = \pm(\omega_1 - \omega_2)$. When the difference frequency is not large, this term will appear close to the DC band. Therefore, there will be side peaks in the DC band for a SF-generated signal. This can be seen clearly in the simulated result (Supplementary Fig. 14d), which also explains the side peaks in the DC band of the experimental data of BBO (Supplementary Fig. 4b) and the nanosponges (Supplementary Figs. 11 and 12). When inspecting the last three interference terms in the intensity expression in Eq. (21), we see that they have similar forms of their modulation along the time delay axis τ. Therefore, their Fourier transform along τ will give similar delay frequency dependencies as those derived above, but the signal appears at different detection frequencies determined by the line shape function along ω_d. This does not affect the conclusions drawn from the inspection of the DC and FM band terms discussed above.

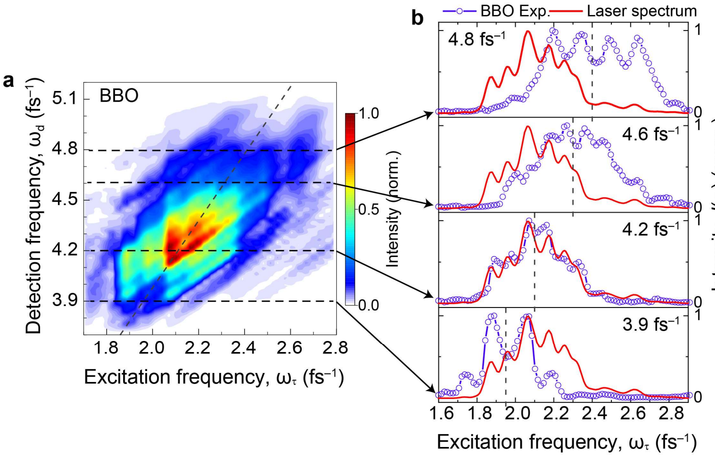

Supplementary Fig. 15 | FM band of IFRAC traces recorded with a BBO sample. (a) Fourier-transformed FM band of the IFRAC trace from a BBO sample shown in Supplementary Fig. 4c. The signal is symmetric with respect to the diagonal dashed line with a slope of 2, indicating a 2nd-order nonlinear process. A splitting of the signal, symmetric with respect to the diagonal, is observed throughout the entire emission range and is the signature of SF generation ($\omega_d = \omega_1 + \omega_2$) from the broad laser spectrum. (b) Cross-sectional spectra taken at four different emission frequencies. These spectra, in particular their peak structures, match reasonably well to the laser spectrum, i.e., the excitation spectrum. The cross-sectional spectra are symmetric with respective to $\omega_\tau = \omega_d/2$ (vertical dashed lines in each panel), in accordance with Eq. (26). This is because in SF, the same number of photons at ω_1 and ω_2 are annihilated, symmetrizing the cross-sectional spectra. The result also suggests that the 2nd-order nonlinear emission from BBO excited by a broadband laser essentially results from many SF pathways mixing different frequency components of the laser.

Supplementary Note 8: Rayleigh scattering of nonlinear plasmonic field by ZnO inclusions-analysis of the enhancement factor

In the phenomenological model for the nonlinear plasmon-exciton coupling introduced in the Methods section, we have provided a rough estimate for the enhancement of nonlinear plasmonic field due to their Rayleigh scattering by ZnO inclusion. Eq. (12) in the Methods section shows that, in a simplified coupled point-dipole model, the enhancement factor simply depends on the frequency dependent dielectric function of ZnO, $\varepsilon(\omega) = n(\omega)^2$, with $n(\omega)$ being the refractive index of ZnO. Since we detect the nonlinear signal from the hybrid nanosponge in a broad emission range, we are sensitive to the spectral variation of enhancement at different

frequencies and, in particular, to the resonant enhancement near the ZnO bandgap. The excitonically-enhanced on-resonant refractive index of ZnO near the bandgap is $n^{\mathrm{on}}(\omega_{\mathrm{X}} = 4.8\ \mathrm{fs}^{-1}) = 2.3$,[18] gives $\varepsilon^{\mathrm{on}}(\omega_{\mathrm{X}}) = 5.3$ and an upper limit of the on-resonant enhancement of $F_{\mathrm{enh}}^{\mathrm{on}} \approx \left|3\varepsilon/(\varepsilon+2)\right|^2 = 4.7$. The off-resonant refractive index at the main nonlinear plasmonic peak at ω = 4.4 fs^{-1} is $n^{\mathrm{off}}(\omega) = 2.1$,[18] giving $\varepsilon^{\mathrm{off}}(\omega) = 4.4$, and thus $F_{\mathrm{enh}}^{\mathrm{off}} = 4.25$. The ratio between the on- and off-resonant enhancement factors is thus $R = F_{\mathrm{enh}}^{\mathrm{on}} / F_{\mathrm{enh}}^{\mathrm{off}} \sim 1.1$. This means that the on-resonant enhancement at ω_{X} is expected to be ~10% stronger than the off-resonant enhancement. Such a small ratio cannot explain the experimentally observed large enhancement at ω_{X} that is seen in Fig. 4a of the main manuscript and in Supplementary Fig. 6. Note that F should be taken as an upper limit for the enhancement factor since i) it is assumed that the effective dipole moment of the nonlinear plasmonic field is directly located at the surface of the ZnO inclusion, ii) the two dipoles are aligned in parallel, and iii) the phases of the two dipoles are identical.

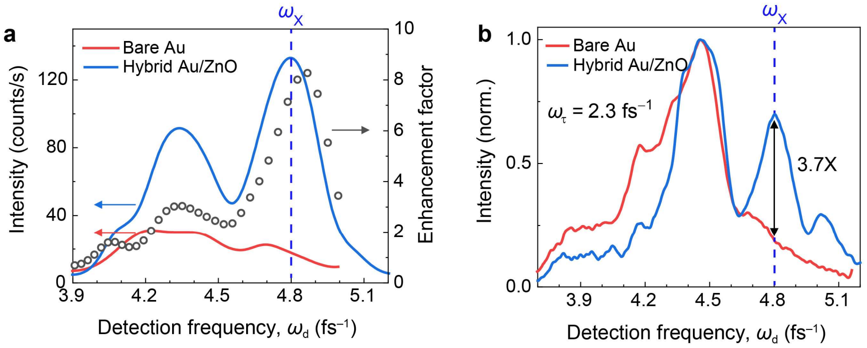

Supplementary Fig. 16 | Emission frequency dependent enhancement factor. (a) Spectral envelope of the nonlinear emission of bare Au and hybrid Au/ZnO nanosponges, taken from the polarization-resolved measurements in Supplementary Figs. 5 and 6 by Fourier filtering. The data for Au/ZnO nanosponge (light blue) is recorded for a fixed polarization angle of the incident light at θ = 110° (Supplementary Fig. 6d). At this angle, the strongest emission intensity is observed near the ZnO bandgap. The data for the bare Au nanosponge (red) shows a polarization-averaged spectrum, obtained from the results shown in Supplementary Fig. 5b. An enhancement factor of ~2 is observed in the range of off-resonant emission frequencies, ω_{d} = 4.0–4.6 fs^{-1} (open circles). This enhancement factor increases to ~8 for emission near the ZnO bandgap, resonant to the exciton frequency ω_{X}. (b) Vertical cross-sections taken at ω_τ = 2.3 fs^{-1} from the FM band of the IFRAC traces recorded on a bare Au and a hybrid Au/ZnO nanosponge that are shown in Supplementary Fig. 7. The tail below ω_{d} = 4.4 fs^{-1} in the bare Au data is a TPL signal. The ratio between on- and off-resonant enhancement factor is $R \approx 3.7$, see detailed discussion in the text.

Experimentally, it is not straightforward to get an unambiguous value for the enhancement factor because it is difficult to perform measurements on the same nanosponge before and after the infiltration of ZnO. Our measurements are therefore performed on nanosponges with similar sizes, to allow for a reasonably good estimate. We find that, for bare gold nanosponges, the nonlinear emission spectra and their intensity do not vary much with incident polarization (Supplementary Fig. 5). We thus take the envelope of the spectrum obtained after averaging over all incident polarization angles as a reference signal for the bare gold nanosponge (red curve, Supplementary Fig. 16a). For the hybrid Au/ZnO nanosponge, the intensity of emission spectra near the ZnO bandgap depends sensitively on the incident polarization. We take the

spectrum with the strongest exciton peak (blue curve, Supplementary Fig. 16a) because this reflects an efficient excitation of hot spots that align well and thus couple strongly to ZnO excitons, which is the desired condition for exploring such coupling effects. Comparing the intensities of these two spectra gives detection frequency dependent enhancement factors, $F(\omega) = I_{\mathrm{Au/ZnO}}(\omega) / I_{\mathrm{Au}}(\omega)$, as shown in Supplementary Fig. 16a as open circles. The enhancement factor is ~8 at ω_X, near the ZnO bandgap. This is close to other SH or third-harmonic enhancement factors of about one order of magnitude that have been reported in the literature, when placing nonlinear dielectrics ZnO[19] and indium tin oxide (ITO)[20] inside the localized modes of plasmonic nanoresonators.

It is seen from Supplementary Fig. 16a that the ratio between on-and off-resonant enhancement, R ~ 4. It is thus much higher than from the above estimate (~1.1) for the resonant Rayleigh scattering of plasmonic SH by the ZnO inclusion. A similar value for the ratio R can also be estimated from the FM band, which is more sensitive to the effect of the selective excitation of certain hot spots. For that, we take vertical cross-sectional spectra $I(\omega_{\mathrm{d}}, \omega_\tau = 2.3\ \mathrm{fs}^{-1})$ through the FM band shown in Supplementary Fig. 7 at a fixed delay frequency of $\omega_\tau = 2.3\ \mathrm{fs}^{-1}$. This is the excitation frequency that leads to a strong resonant enhancement of the nonlinear emission at exciton frequency. A comparison of this cross-sectional spectra from the hybrid Au/ZnO nanosponge to the one obtained from the bare gold nanosponge is shown in Supplementary Fig. 16b. The spectra are normalized to the maximum off-resonant emission intensity. They reveal an on-resonant enhancement ratio of $R \approx 3.7$, similar to that seen in Supplementary Fig. 16a. Such an on-resonant enhancement ratio is difficult to reconcile with the 2nd-order coupling pathway, i.e., the Rayleigh scattering of nonlinear plasmonic field by the ZnO inclusion. Since the linear dielectric function of ZnO does not vary much around the ZnO bandgap, the coupled-dipole model outlined in the Methods predicts a mostly frequency independent enhancement factor, which is in stark contrast to the experiment. Our analysis thus suggests that the dominant enhancement arises from the plasmon-driven nonlinear emission from ZnO, using its larger 2nd-order susceptibility that is resonantly enhanced near the band gap.[21-23]

Supplementary Note 9: Simulation using a nonlinear coupled-oscillator model

Exclusion of direct far-field laser excited nonlinear excitonic emission from ZnO

To simulate pure far-field laser excited nonlinear emission from ZnO, only laser field $E_{\mathrm{L}}(t)$ is used as the excitation source to drive the ZnO excitonic oscillator in Eq. (6) in the Methods section. The simulated results are shown in Supplementary Fig. 17. The IFRAC trace is close to our experimental result measured on ZnO nanostructures,[11] showing a pronounced emission peak at exciton frequency. Its FM band (Supplementary Fig. 17b) is very different from the experimental data of the hybrid nanosponge shown in Fig. 4a in the main text. Particularly, the cross-sectional spectrum $I(\omega_{\mathrm{d}} = \omega_{\mathrm{X}}, \omega_\tau)$ at the exciton frequency, is much broader than that of the experimental data in Fig. 4a, covering almost the entire bandwidth of the laser (Supplementary Fig. 17c). This broad line shape is due to an efficient sum-frequency excitation of the ZnO exciton by correlated frequency components $\omega_1 + \omega_2 = \omega_{\mathrm{X}}$ of the laser spectrum. We thus exclude that the nonlinear excitonic emission in the hybrid nanosponge is excited by far-field laser.

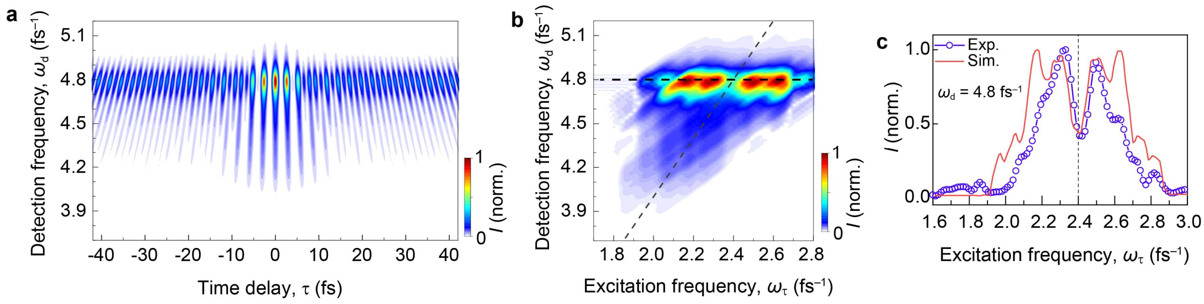

Supplementary Fig. 17 | Simulation of nonlinear emission from ZnO with direct far-field laser excitation. (a) Simulated IFRAC trace. (b) FM band of the simulated IFRAC trace. The diagonal line has a slope of 2. (c) Cross-sectional spectrum $I(\omega_\tau)$ (red solid line) taken at $\omega_d = 4.8$ fs^{-1} from the FM band as marked by the horizontal line in (b), which is much wider than the experimental spectrum (blue open circles) from the hybrid nanosponge shown in Fig. 4a of the main text.

Phase of the three pathways contributing to nonlinear emission from hybrid nanosponges

In the manuscript, we have discussed three pathways that contribute to the nonlinear emission from hybrid nanosponges: (a) nonlinear emission from plasmonic hot spots; (b) plasmon-enhanced nonlinear excitonic emission from ZnO (1st-order coupling); and (c) Rayleigh scattering of nonlinear plasmonic field by ZnO inclusion (2nd-order coupling). Here, we analyze the phase of the emitted fields from these different pathways by taking the phase of the incident laser field as ϕ_L.

(a) Nonlinear plasmonic dipole moment $p_{Au}^{(2)}$: the resonant linear plasmonic dipole moment $p_{Au}^{(1)}$ has a phase $\phi_L + 90°$ (Eq. (4) in Methods) when it is resonantly driven. Its square, with a phase of $2\phi_L + 180°$, drives the off-resonant, low-frequency plasmon mode (Eq. (5) in Methods). This leads to a 180° phase shift, giving a phase of $2\phi_L$ for the nonlinear plasmonic dipole moment $p_{Au}^{(2)}$.

(b) Nonlinear excitonic dipole moment $p_{ZnO}^{(2)}$ via 1st-order coupling: here, the linear plasmon dipole $p_{Au}^{(1)}$ ($\phi_L + 90°$) off-resonantly drives the high-frequency exciton mode (Eq. (6) in Methods), giving an in-phase linear response of the exciton, i.e., the phase is still $\phi_L + 90°$. In 2nd-order perturbation (Eq. (7) in Methods), its square, with a phase of $2\phi_L + 180°$, resonantly drives the exciton oscillator (90° phase shift), leading to a phase $2\phi_L + 270°$ for the nonlinear excitonic dipole moment $p_{ZnO}^{(2)}$. This is –90° phase-shifted relative to $p_{Au}^{(2)}$, as seen from the simulated fields shown in Fig. 4d in the main text.

(c) Linear exciton dipole moment $p_{ZnO}^{(1)}$ via 2nd-order coupling: as discussed in Methods, there are two contributions: (i) the resonant scattering of $p_{Au}^{(2)}$ by the exciton. This resonant interaction (Eq. (6) in Methods) leads to a 90° phase shift. The resulting phase of the scattered field of the dipole moment $p_{ZnO}^{(1)}$ is then $2\phi_L + 90°$, that is shifted by 90°relative to $p_{Au}^{(2)}$, but 180° phase shifted relative to $p_{ZnO}^{(2)}$; (ii) the field that is scattered by the real-valued, off-resonant dielectric function of the ZnO inclusion, which is in phase with $p_{Au}^{(2)}$. In the case of ZnO, the total scattered field is dominated by the large background dielectric function contribution, and is thus almost in-phase with $p_{Au}^{(2)}$, as seen from the simulated fields shown in Fig. 4d in the main text.

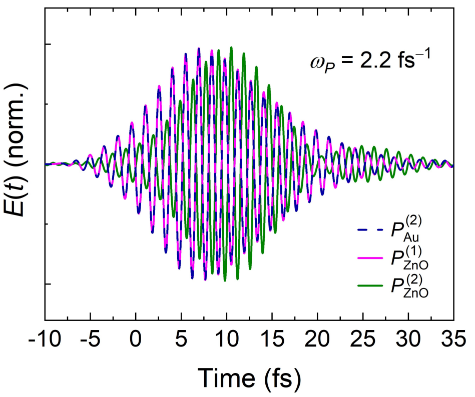

Supplementary Fig. 18 | Simulated electric fields of the three emission pathways. For a low-frequency hot spot with $\omega_P = 2.2$ fs^{-1}, the nonlinear plasmon field and nonlinear exciton fields are almost out-of-phase. Since the material is off-resonantly excited, the time structure of the nonlinear exciton field is also not stretched in time, in stark contrast to the result in Fig. 4d for the hot spot at $\omega_P = 2.5$ fs^{-1}. The linear scattering field by ZnO is in-phase with the nonlinear plasmonic field and has almost the same time profile because of the dominant off-resonant ε_B contribution.

For a plasmonic hot spot mode centered at $\omega_P = 2.5$ fs^{-1}, the phase of these three pathways can be seen in the simulated electric fields in Fig. 4d in the main text. We note, however, that if the nonlinear plasmonic frequency is lower than that of the exciton resonance, the nonlinear excitonic dipole moment $p^{(2)}_{\mathrm{ZnO}}$ will have a phase $2\phi_L + 180°$ (because the phase-shift will be 0° for Eq. (7) in Methods). The nonlinear plasmonic and excitonic emissions will thus be out-of-phase, as shown for the simulation of hot spot with $\omega_P = 2.2$ fs^{-1} in Supplementary Fig. 18. This further reduces the overall emission efficiency for coupling low-frequency ($\omega_P < \omega_X / 2$) hot spots to excitons.

Simulation of IFRAC traces resulting from the coupling between multiple hot spots and excitons

A realistic modelling for the nonlinear emission from the hybrid nanosponge should come from the coupling of ZnO to a series of different, randomly disordered plasmonic hot spot modes. We try to implement this in the simulations of the experimental IFRAC results by including this ensemble of plasmonic hot spots in the nonlinear plasmon-exciton coupling model. For this, we assume 7 evenly distributed hot spot modes resonant at $\omega_P = 2.0$–2.6 fs^{-1} with frequency spacing $\Delta\omega = 0.1$ fs^{-1} and the same damping rate of $\gamma_P = 0.06$ fs^{-1}. We have observed in Supplementary Fig. 6 that a rotation of the polarization of the incident pulse leads to different enhancements of the excitonic emission, reflecting the excitation of different hot spot modes with different weights. The amplitude of each mode is thus adjusted to achieve reasonable agreement between simulation and the experimental IFRAC trace (Supplementary Fig. 19a). The exciton resonance is set at $\omega_X = 4.8$ fs^{-1} with $\gamma_X = 0.08$ fs^{-1}, matching the values from photoluminescence measurements.[12, 19, 24] Since the external laser excitation is known to be inefficient, only plasmonic hot spot fields $x^{(1)}$ are used as the driving term in Eq. (6) in Methods. In the simulation, e/m is taken as a constant of one, and the other parameters are: $a_P = 1$, $a_X = 0.25$, $\Omega_1 = 1$, $\Omega_2 = 1$. The relative weights of the three pathways are chosen as $a = 0.2$, $b = 10$, $c = 0.2$. Note that a variation of these parameters does not significantly affect the main features of the IFRAC trace, time dynamics, and FM band structure. It mostly affects the relative enhancement factors of the nonlinear emission from the hybrid nanosponge, providing a means

to approximate the experimental results by the simulations. With the chosen parameters, for hybrid Au/ZnO nanosponge, the excitonic emission integrated between 4.6 –5.0 fs^{-1} is roughly one order of magnitude stronger than that of the bare gold particle (~40 times in our simulation), but at least two to three-orders of magnitude stronger than that of bare ZnO (~1350 times in our simulation), consistent with literature reports.[19] Supplementary Fig. 19b shows the simulated nonlinear emission spectra in which a distinct excitonic emission peak is observed. The simulated IFRAC traces (Supplementary Fig. 19c), FM band (Fig. 4b in the main text), IAC traces (Fig. 3d in the main text), and the cross-sectional spectrum at the exciton emission frequency (Supplementary Fig. 19d) all reproduce well the results of our experiments.

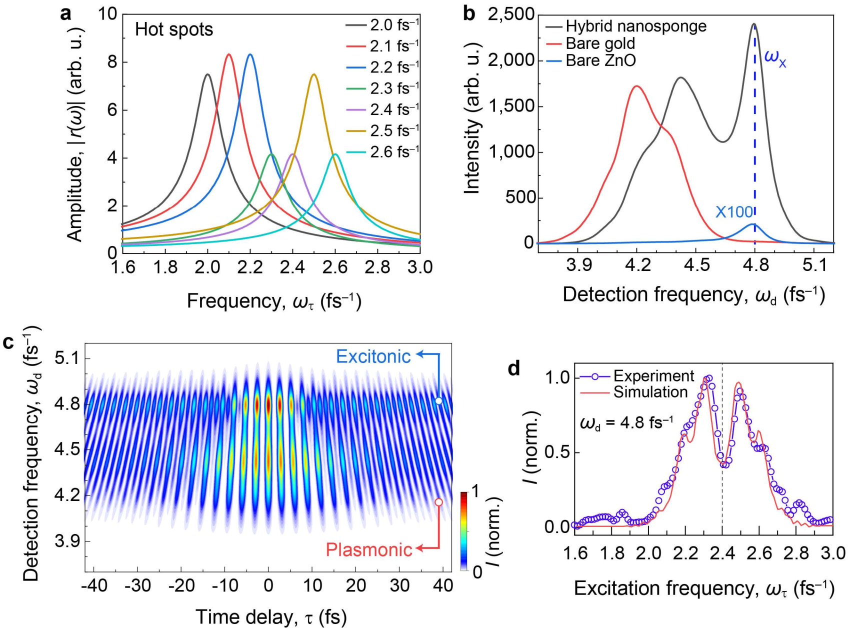

Supplementary Fig. 19 | Simulation of enhanced excitonic emission by nonlinear coupling to multiple plasmonic hot spots. (a) Response functions of 7 evenly distributed hot spot resonances with different amplitudes. (b) Simulated nonlinear emission spectra of a hybrid nanosponge, a bare gold nanosponge, and a bare ZnO layer. A distinct emission enhancement at the exciton frequency is observed which is good agreement with the measured spectrum in Supplementary Fig. 16. (c) Simulated IFRAC trace of the hybrid nanosponge, agreeing reasonably well with the experiment shown in Fig. 3b in the main text. (d) Cross-sectional spectrum $I(\omega_d = \omega_X, \omega_\tau)$ taken at $\omega_d = 4.8$ fs^{-1} from the simulated FM band shown in Fig. 4b in the main text (red solid line). The spectrum matches well with the experimental data taken from the FM band in Fig. 4a in the main text (blue open circles). The IAC trace of this simulation is shown in Fig. 3d in the main text.

Effect of detuning of hot spots on the nonlinear plasmon-exciton coupling

The simulated result shown above are obtained by considering the coupling between excitons and multiple plasmonic hot spots. This ensemble of hot spots gives rise to a spectrally broadband plasmonic SH emission. The 2D-FM spectrum reveals also the coupling of each of these frequency components to the ZnO exciton resonance and this allows us to study the effect of hot spot detuning on the plasmon-exciton coupling. To analyze this effect in more detail, we have performed simulations individually for each hot spot with the same parameters used above. The results are shown in Supplementary Fig. 20.

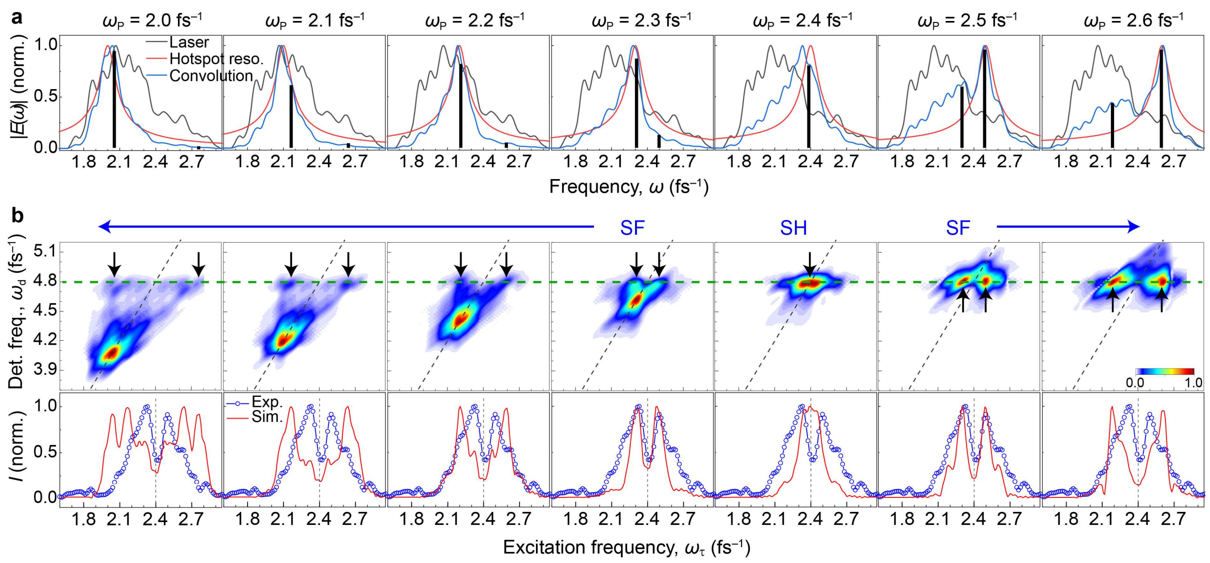

Supplementary Fig. 20 | Effect of detuning of hot spots on the nonlinear plasmon-exciton coupling. (a) Field amplitudes $|E(\omega)|$ of laser (gray), response function of the hot spot resonance (red), and the convoluted plasmonic hot spot field (blue) simulated for each of the 7 hot spots (the resonance frequencies are indicated on top). The plasmonic hot spot fields contain peak structures originating from the substructures of the broadband laser field due to their linear convolution. The black lines mark the frequency components at $\omega_1 \sim \omega_P$ and $\omega_2 \sim \omega_X - \omega_P$ and their amplitudes, reflected by the height of the black lines, that couple to the exciton mode by SF or SH generation. (b) Top row: FM bands simulated for the coupling of a single hot spot resonance to the exciton mode for each of the 7 hot spots. The diagonal dashed line has a slope of 2. The horizontal green dashed line marks the excitonic emission frequency. The black arrows depict the main fundamental field components that couple to exciton via SH or SF mixing. Bottom row: Cross-sectional spectra $I(\omega_d = \omega_X, \omega_\tau)$ taken at $\omega_d = 4.8$ fs^{-1} (red solid lines) from the simulated FM bands, compared to the experimental data taken from the FM band in Fig. 5a in the main text (blue open circles).

After laser excitation, the plasmonic hot spot fields are given as the linear convolution between the laser field and the response function of the hot spots and thus contain the sub-peak structures from the laser spectrum (Supplementary Fig. 20a). Simulated FM bands for the coupling of a single hot spot to the ZnO exciton mode are shown in Supplementary Fig. 20b (top row). In general, a main plasmonic emission peak is observed at $\omega_d \approx 2\omega_P$, particularly for the low-frequency hot spots. Additionally, an excitonic emission peak at $\omega_d \sim \omega_X$ can be observed as marked by the horizontal green dashed line. An analysis of the FM bands shows that the excitonic emission can be generated via SH generation for the hot spot at $\omega_P = 2.4$ fs^{-1}, i.e., $\omega_X/2$. This SH generation, however, does not match to the experimental data, suggesting that this hot spot is not efficiently excited due to the dip structure at 2.4 fs^{-1} in the spectrum of our laser. Alternatively, excitonic emission can be generated via SF channels for the other detuned hot spots, as marked by black arrows in each image. For SF generation, the two main linear field components are centered at $\omega_1 \approx \omega_P$ and $\omega_2 \approx \omega_X - \omega_P$ to fulfil energy conservation. This is more clearly observed in the cross-sectional spectra $I(\omega_d = \omega_X, \omega_\tau)$ taken at $\omega_d = 4.8$ fs^{-1} from each of the FM bands (Supplementary Fig. 20b, bottom row). A comparison to the experimental data suggests that the excitonic emission is mainly enhanced by coupling the plasmonic hot spots at either $\omega_P = 2.3$ fs^{-1} or $\omega_P = 2.5$ fs^{-1}. In both cases, this generates peaks $\omega_\tau = 2.3$ fs^{-1} and $\omega_\tau = 2.5$ fs^{-1} in the 2D-FM spectrum due to SF generation. The cross-sectional spectra of these

two hot spots, however, cannot fully account for the experimental data, indicating the coupling of exciton also to other hot spots. For example, the cross-sectional spectrum for the $\omega_P = 2.2$ fs^{-1} hot spot gives better agreements for the more detuned frequency parts. We thus conclude that the excitonic emission is driven by multiple hot spots in the nanosponge. The more detuned hot spots couple weaker to the exciton because the strength of the nonlinear excitonic polarization, $P_{Zno}^{(2)} \propto E_P(\omega_1)E_P(\omega_2)$, scales with the product of the amplitudes of the two plasmonic field components. The larger the detuning of the hot spot, the weaker the other frequency component, as marked by black lines in Supplementary Fig. 20a. We finally point out that the two frequency components for SF mixing do most probably not arise from simultaneous coupling of a ZnO inclusion to two individual hotspots. This is because the hotspots are well localized in space without mode overlap.[13] The linear plasmonic field of two spatially well-separated hotspots are therefore unlikely to be coherently interact with the same ZnO inclusion. Instead, the two 'modes' ω_1 and ω_2 come from a single hotspot upon impulsive excitation with the broadband, few-cycle laser as discussed here.

Supplementary Note 10: Fitting of the PEEM data

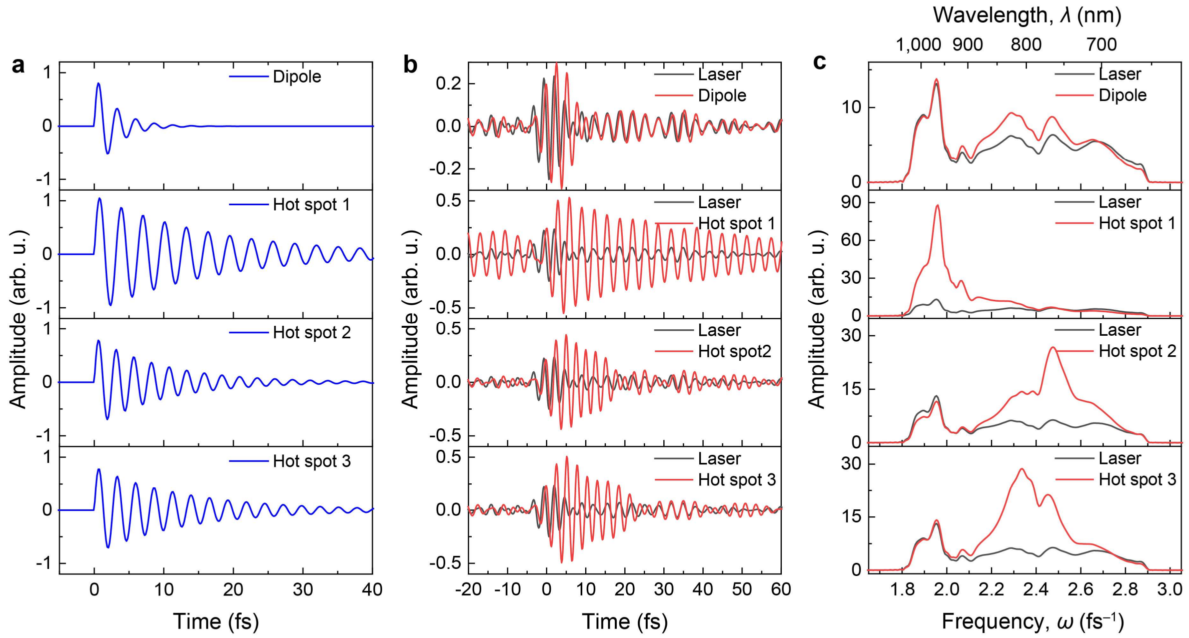

Supplementary Fig. 21 | Response functions of dipole and hot spot modes for the fitting of PEEM data. (a) Time-domain response functions of the plasmonic dipole mode and hot spot modes. The parameters of those plasmon resonances are discussed in Methods section in the main text. (b) Electric field amplitudes of the laser (black) and its convolution with the dipole and hot spot modes (red) in time domain. (c) Fourier transforms of the time domain signals to frequency domain, showing relatively weak, broadband response of the dipole mode and strong, spectrally narrow responses of the hot spot modes.

Supplementary References

1. Wang, D. & Schaaf, P. Nanoporous gold nanoparticles. *J. Mater. Chem.* **22**, 5344-5348 (2012).
2. Rao, W. et al. Nanoporous Gold Nanoparticles and Au/Al2O3 Hybrid Nanoparticles with Large Tunability of Plasmonic Properties. *ACS Appl. Mater. Interfaces* **9**, 6273-6281 (2017).
3. Wang, D. & Schaaf, P. Plasmonic nanosponges. *Adv. Phys. X* **3**, 1456361 (2018).
4. Vidal, C., Wang, D., Schaaf, P., Hrelescu, C. & Klar, T.A. Optical Plasmons of Individual Gold Nanosponges. *ACS Photonics* **2**, 1436-1442 (2015).
5. Hergert, G. et al. Long-lived electron emission reveals localized plasmon modes in disordered nanosponge antennas. *Light: Sci. App.* **6**, e17075 (2017).
6. Vidal, C. et al. Plasmonic Horizon in Gold Nanosponges. *Nano Lett.* **18**, 1269-1273 (2018).
7. Stibenz, G. & Steinmeyer, G. Interferometric frequency-resolved optical gating. *Opt. Exp.* **13**, 2617-2626 (2005).
8. Hyyti, J., Escoto, E. & Steinmeyer, G. Third-harmonic interferometric frequency-resolved optical gating. *J. Opt. Soc. Am. B* **34**, 2367-2375 (2017).
9. Hyyti, J., Escoto, E. & Steinmeyer, G. Pulse retrieval algorithm for interferometric frequency-resolved optical gating based on differential evolution. *Rev. Sci. Instrum.* **88**, 103102 (2017).
10. Kane, D.J. & Trebino, R. Characterization of arbitrary femtosecond pulses using frequency-resolved optical gating. *IEEE J. Quantum Electron.* **29**, 571-579 (1993).

11. Mascheck, M. et al. Observing the localization of light in space and time by ultrafast second-harmonic microscopy. *Nat. Photon.* **6**, 293-298 (2012).
12. Schmidt, S. et al. Distinguishing between ultrafast optical harmonic generation and multi-photon-induced luminescence from ZnO thin films by frequency-resolved interferometric autocorrelation microscopy. *Opt. Exp.* **18**, 25016-25028 (2010).
13. Zhong, J. et al. Strong Spatial and Spectral Localization of Surface Plasmons in Individual Randomly Disordered Gold Nanosponges. *Nano Lett.* **18**, 4957-4964 (2018).
14. Stockman, M.I., Bergman, D.J., Anceau, C., Brasselet, S. & Zyss, J. Enhanced Second-Harmonic Generation by Metal Surfaces with Nanoscale Roughness: Nanoscale Dephasing, Depolarization, and Correlations. *Phys. Rev. Lett.* **92**, 057402 (2004).
15. Klein, M.W., Tritschler, T., Wegener, M. & Linden, S. Lineshape of harmonic generation by metallic nanoparticles and metallic photonic crystal slabs. *Phys. Rev. B* **72**, 115113 (2005).
16. Anceau, C., Brasselet, S., Zyss, J. & Gadenne, P. Local second-harmonic generation enhancement on gold nanostructures probed by two-photon microscopy. *Opt. Lett.* **28**, 713-715 (2003).
17. Bozhevolnyi, S.I., Beermann, J. & Coello, V. Direct Observation of Localized Second-Harmonic Enhancement in Random Metal Nanostructures. *Phys. Rev. Lett.* **90**, 197403 (2003).
18. Sun, X.W. & Kwok, H.S. Optical properties of epitaxially grown zinc oxide films on sapphire by pulsed laser deposition. *J. App. Phys.* **86**, 408-411 (1999).
19. Grinblat, G. et al. High-Efficiency Second Harmonic Generation from a Single Hybrid ZnO Nanowire/Au Plasmonic Nano-Oligomer. *Nano Lett.* **14**, 6660-6665 (2014).
20. Aouani, H., Rahmani, M., Navarro-Cía, M. & Maier, S.A. Third-harmonic-upconversion enhancement from a single semiconductor nanoparticle coupled to a plasmonic antenna. *Nat. Nanotech.* **9**, 290 (2014).
21. Larciprete, M.C. & Centini, M. Second harmonic generation from ZnO films and nanostructures. *App. Phys. Rev.* **2**, 031302 (2015).
22. Zhang, X.Q., Tang, Z.K., Kawasaki, M., Ohtomo, A. & Koinuma, H. Resonant exciton second-harmonic generation in self-assembled ZnO microcrystallite thin films. *J. Phys.: Condens. Matter* **15**, 5191-5196 (2003).
23. Lafrentz, M. et al. Second-harmonic generation spectroscopy of excitons in ZnO. *Phys. Rev. B* **88**, 235207 (2013).
24. Hyun, J.K. et al. Enhanced Second Harmonic Generation by Coupling to Exciton Ensembles in Ag-coated ZnO Nanorods. *ACS Photonics* **2**, 1314-1319 (2015).
