# Supplementary material for: Nonlinear plasmon-exciton coupling enhances sum-frequency generation from a hybrid metal/semiconductor nanostructure
Source: Nat Commun. 2020 Mar 19;11:1464. doi: 10.1038/s41467-020-15232-w (PMC7081225; doi:10.1038/s41467-020-15232-w)
Supplement: Supplementary file 5 — Description of Additional Supplementary Files [file 41467_2020_15232_MOESM5_ESM.pdf]

Description of Additional Supplementary Files

Title: Supplementary Movie 1
Description: Inter-pulse time delay dependent microscopic photoelectron emission images for the gold nanosponge shown in Figure 2.

Title: Supplementary Movie 2
Description: Inter-pulse time delay dependent microscopic photoelectron emission images for other gold nanosponges.
